# Offering mental health first aid to a person with depression: a Delphi study to re-develop the guidelines published in 2008

**DOI:** 10.1186/s40359-019-0310-3

**Published:** 2019-06-21

**Authors:** Kathy S. Bond, Fairlie A. Cottrill, Fiona L. Blee, Claire M. Kelly, Betty A. Kitchener, Anthony F. Jorm

**Affiliations:** 1 Mental Health First Aid Australia, Parkville, Victoria Australia; 2 Centre for Mental Health, Melbourne School of Population and Global Health, University of Melbourne, Parkville, Victoria Australia; 3 Department of Psychology, Faculty of Health, Deakin University, Burwood, Victoria Australia

**Keywords:** Depression, Mental health first aid, Delphi study

## Abstract

**Background:**

Depressive disorder is ranked as the largest contributor to non-fatal health burden. However, with prompt treatment, outcomes can improve. Family and friends are well placed to recognise the signs of depression and encourage early help seeking. Guidelines about how members of the public can provide mental health first aid to someone who is experiencing depression were developed in 2008. A Delphi study was conducted to re-develop these guidelines to ensure they are current and reflect best practice.

**Methods:**

A survey was developed using the 2008 depression mental health first aid guidelines and a systematic search of grey and academic literature. The questionnaire contained items about providing mental health first aid to a person with depression. These items were rated by two international expert panels – a lived experience panel (consumers and carers) and a professional panel.

**Results:**

Three hundred and fifty-two items were rated by 53 experts (36 with lived experience and 17 professionals) according to whether they should be included in the revised guidelines. There were 183 items that met the criteria to be included in the updated guidelines.

**Conclusions:**

This re-development has added detail to the previous version of the guidelines, giving more guidance on the role of the first aider and allowing for a more nuanced approach to providing first aid to someone with depression. These guidelines are available to the public and will be used to update the Mental Health First Aid courses.

**Electronic supplementary material:**

The online version of this article (10.1186/s40359-019-0310-3) contains supplementary material, which is available to authorized users.

## Background

In 2015 it was estimated that 4.4% of the world’s population experienced a depressive disorder in the past year, and these disorders were ranked as the largest contributor to non-fatal health burden [1]. If depression is not treated promptly, outcomes tend to be worse and the person is more likely to have subsequent and worse episodes of depression [1, 2].

Family and friends are well placed to recognise the signs of depression and assist a person with depression to get early help. While the public’s knowledge about depression is higher than for other mental health conditions, such as anxiety disorder and psychosis [3], this does not necessarily translate into knowing what actions to take to support a person with depression [4]. For this reason, the Mental Health First Aid (MHFA) course was developed [5]. The course teaches adults how to recognise when someone is developing a mental health problem or crisis and to assist them by offering mental health first aid. Similar to physical first aid, mental health first aid is offered by members of the public to their friends, family, co-workers, etc. and is defined as [6]:

*The help offered to a person developing a mental health problem, experiencing a worsening of an existing mental health problem or in a mental health crisis. The first aid is given until appropriate professional help is received or until the crisis resolves.*

The MHFA course has been extensively evaluated and shown to improve knowledge about mental health problems, the ability to recognise a mental health problem and confidence in the ability to help a person with a mental health problem [7].

The content of this course is based on a series of expert consensus guidelines developed using the Delphi method (e.g. [8, 9]), including guidelines on how to provide mental health first aid for depression, developed in 2008 [10]. These guidelines were used to inform the content of the 2nd, 3rd and 4th editions of the Australian MHFA course, which is the parent of MHFA courses internationally [5, 11, 12]. These guidelines are available on the MHFA Australia website. The usefulness of these guidelines to people who download them from the website was evaluated by Hart and colleagues [13]. They found that the guidelines contributed to a meaningful conversation about the person’s mental health problems, and in some cases the person sought professional help. The users of the guidelines stated they were able to assist in a way that was knowledgeable and supportive. The guidelines are a general set of recommendations, and because each person is unique, the guidelines may not be suitable to every situation. However, they are designed to be useful for most people, most of the time. To ensure that the guidelines are current and reflect best practice, they are updated on a regular schedule, similar to clinical practice guidelines being regularly updated (e.g. [14]). With the MHFA Australia guidelines, this re-development is carried out at least every 10 years, using the Delphi method. The mental health first aid guidelines for suicidal thoughts and behaviours, and non-suicidal self-injury were the first guidelines to be revised using the Delphi method and significant revisions were indicated, specifically a number of more detailed and specific first aid actions were recommended [15, 16] further justifying the need to regularly revise the full suite of guidelines.

The Delphi method is a systematic way of determining expert consensus [17] and it is often used to develop guidelines using practice-based evidence. It is considered an ethical and feasible way to develop guidelines on a topic that is not amenable to evaluation using other methods, e.g. randomised controlled trials. The method can be implemented online, allowing expert consensus to be obtained from participants located in many countries. Development of the current guidelines followed the protocol of similar Delphi studies conducted on topics such as mental health first aid guidelines for non-suicidal self-injury and assisting Australians with mental health problems and financial difficulties [18].

The aim of this study was to re-develop the 2008 Mental Health First Aid Guidelines for Depression [10] using the Delphi method to ascertain the consensus of international experts from high-income western countries. As expertise on how to give mental health first aid may come from either professional or personal experience, the study required the consensus of panels of consumers, carers and mental health professionals.

## Methods

This Delphi study was conducted in four steps: (1) recruit expert panel members (participants), (2) conduct literature search and develop survey, (3) collect and analyse data and (4) re-develop the 2008 guidelines.

### Step 1: recruit expert panels

People from high-income countries that have licenced the Mental Health First Aid program (Australia, Canada, Denmark, England, Finland, Ireland, The Netherlands, New Zealand, Northern Ireland, Scotland, Sweden, The United States and Wales) were invited to join one of three expert panels: Consumer, Carer or Professional. Researchers aimed to recruit at least 30 participants to each panel to allow for attrition and produce stable results [17].

Participants were recruited by sending a flyer to Australian and international networks, instructors associated with MHFA Australia, and to Australian and international mental health promotion and professional organisations, peak bodies, and advocacy and carer groups. Participants were asked to pass the flyer on to anyone they thought might be interested in participating.

As per previous Delphi studies (e.g. [19]), participants had to be 18 years or older. The specific expert panel selection criteria were:

- Consumer panel – Have a lived experience of depression with the depression being currently well managed **AND** be involved in activities that expose the participant to a broader experience of depression, e.g. advisory or advocacy group, peer support, etc.
- Carer panel – Have experience in providing day-to-day support to someone with depression **AND** be involved in activities that expose the participant to a broader experience of depression, e.g. advisory or advocacy group, peer support, etc.
- Professional panel – have at least 2 years’ experience as a mental health professional or researcher in the field of depression.

### Step 2: literature search and survey development

The first author conducted a literature search of both the ‘grey’ and academic literature in May 2016 to gather statements about how to provide mental health first aid to a person with depression. The literature search was conducted using Google Australia, Google USA, Google UK, Google Books and Google Scholar. Google Scholar was the only academic search engine used because it has a much broader interdisciplinary coverage than other databases and also covers grey academic literature. Our previous experience has been that searches of other databases covering research and professional literature rarely produce information relevant to lay mental health first aid strategies. The key search terms were ‘depression’, ‘clinical depression’, ‘major depressive disorder’, ‘depression carers’, ‘support depression sufferers’ and ‘help depression’. These terms were the terms used in the original Delphi study [20]. The following terms were also included:

- ‘how to help someone with depression’ - generated because this is likely the phrasing a member of the public would use
- ‘major depressive episode’ - generated because this is the term used in DSM 5 diagnostic criteria
- ‘first aid for depression’ - generated because applying the concept of first aid for mental health problems is a more common concept than it was at the time of the first Delphi study.

Based on previous similar Delphi studies [18], the first 50 websites, journal articles and books for each of the search terms were retrieved and reviewed for relevant information. The decision to only examine the first 50 websites, books and journal articles for each search term is based on previous Delphi studies that found that the quality of the resources declined rapidly after the first 50 [21].

**Fig. 1:**
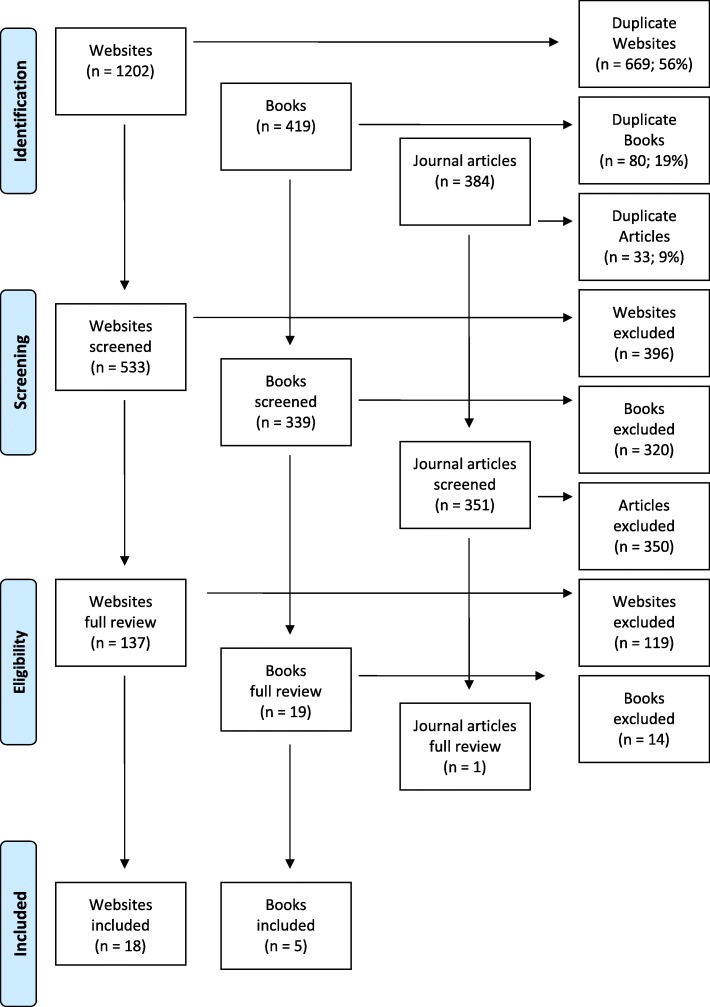
Summary of Literature Search

In order to minimise the influence of Google’s searching algorithms the following steps were taken: signing out of any Google profiles, clearing the search history, disabling location features and deselecting ‘any country’. Links appearing in the websites were reviewed. Websites, articles and books were excluded if they were a duplicate, did not contain information about mental health first aid or were published before the date of the previous Delphi literature search (2007). The content from 137 websites, 19 books and one journal article were analysed to develop the survey with helping statements collated from these sources and reviewed by the research team to ensure that consistent, simple language was used. Figure 1 summarises the literature search results.

**Fig. 2:**
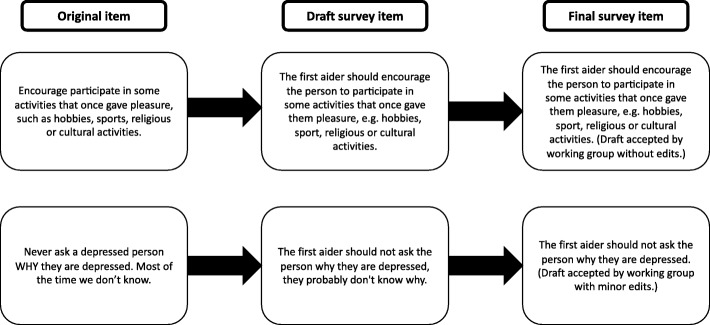
Example of development of survey items

The first author extracted the information from the articles, websites and books and drafted survey items. The research team reviewed the original extracted text and the drafted survey items to finalise them (see Fig. 2 for examples). The survey was administered via SurveyMonkey. Participants rated the survey items, “using a 5-point Likert scale (‘essential’, ‘important’, ‘don’t know/depends’, ‘unimportant’ or ‘should not be included’), according to whether or not they should be included in the guidelines” [22].

### Step 3: data collection and analysis

Between March 2017 and April 2018, data were collected over three rounds of a survey. The Round 1 survey included the survey items developed using the literature search described above and open-ended questions asking for participant comments or suggested new items. The Round 2 survey consisted of these new items and any items needing to be re-rated because they did not receive clear consensus (see point 2 below). The Round 3 survey consisted of items that were new in Round 2 that did not receive clear consensus. See Additional file 1 for copies of the 3 survey rounds.

After participants completed a survey round, the survey items were categorised as follows:

1. Endorsed. The item received an ‘essential’ or ‘important’ rating from at least 80% of participants from each of the panels.
2. Re-rate. The item received an ‘essential’ or ‘important’ rating from 70 to 79% of participants from each of the panels or 80% or more from at least one panel and 70–79% from the remaining panels.
3. Rejected. Item did not meet the criteria to be endorsed or re-rated.

If a re-rated item did not receive an ‘essential’ or ‘important’ rating from 80% or more of participants in each of the panels, it was rejected.

The comments collected in Round 1 were analysed by the working group to develop new items that were not included in the Round 1 survey.

Participants were given a report of Round 1 and 2 responses that included the items that were endorsed, rejected, and the ones that needed to be re-rated in the next Round. For each item that needed to be re-rated, the report included each panel’s percentages for each rating (i.e. “essential”, “important”, etc) and the participant’s individual score. Participants could use this report to compare their ratings with each panel’s ratings and decide if they wanted to change their rating score.

### Step 4: re-develop the 2008 guidelines

The first author wrote the endorsed items into a guidelines document, combining survey items and deleting repetition as needed. However, the original wording was retained as much as possible. Examples and explanatory notes were used for clarification of items. The working group reviewed this draft and it was given to participants for final comment and endorsement.

### Ethics, consent and permissions

This research was approved by the University of Melbourne Human Ethics Committee (ID#1648030). Informed consent, including permission to report individual participant’s de-identified qualitative data, was obtained from all participants by clicking ‘yes’ to a question about informed consent in the Round 1 survey.

## Results

### Participants

Eighty-six people were recruited and 53 completed all three survey rounds (see Table 1 for the retention rate for each of the panels). Of the 53 who completed all three rounds, 38 were females, 14 were males and one person did not wish to disclose their gender. The average age of participants was 46.5 years (SD = 11.61, range 21–69). Participants were from Australia, UK, Ireland, Canada and the USA. The professional panel included educators, researchers, nurses, social workers and psychologists.

**Table 1:** Retention rate

	Round 1	Round 2	Round 3	Retention
Lived Experience	60	38	36	60%
Professional	26	22	17	65%
Total	86	60	53	62%

It was difficult to recruit enough professional and carer experts to allow for stable results. Many of the carers also had professional experience so, with their permission, they were re-allocated to the professional panel. The one carer with no secondary experience was combined with the consumer panel to form a ‘lived experience’ panel. This was deemed reasonable given the high correlations across items between the panels (see Table 2) and is in line with other similar Delphi studies [12].

**Table 2:** Pearson’s correlations across items between panels

Panels	Pearson’s correlation
Consumer and Carer	0.91
Consumer and prof	0.93
Carer and prof	0.90

The lived experience panel included consumers and carers who were members of advocacy groups (e.g. National Alliance of Mental Illness), formal peer support programs (e.g. Flourish Australia) or who had professional experience (e.g. Mental Health First Aid Instructors). Given that Mental Health First Aid Instructors may be very familiar with the contents of the 2008 Guidelines, the number of Instructors allowed to participate was limited to no more than 50%. Forty-two per cent of the Lived Experience and 53% of the Professional panel were Instructors, for a total of 45%.

### Item rating

**Fig. 3:**
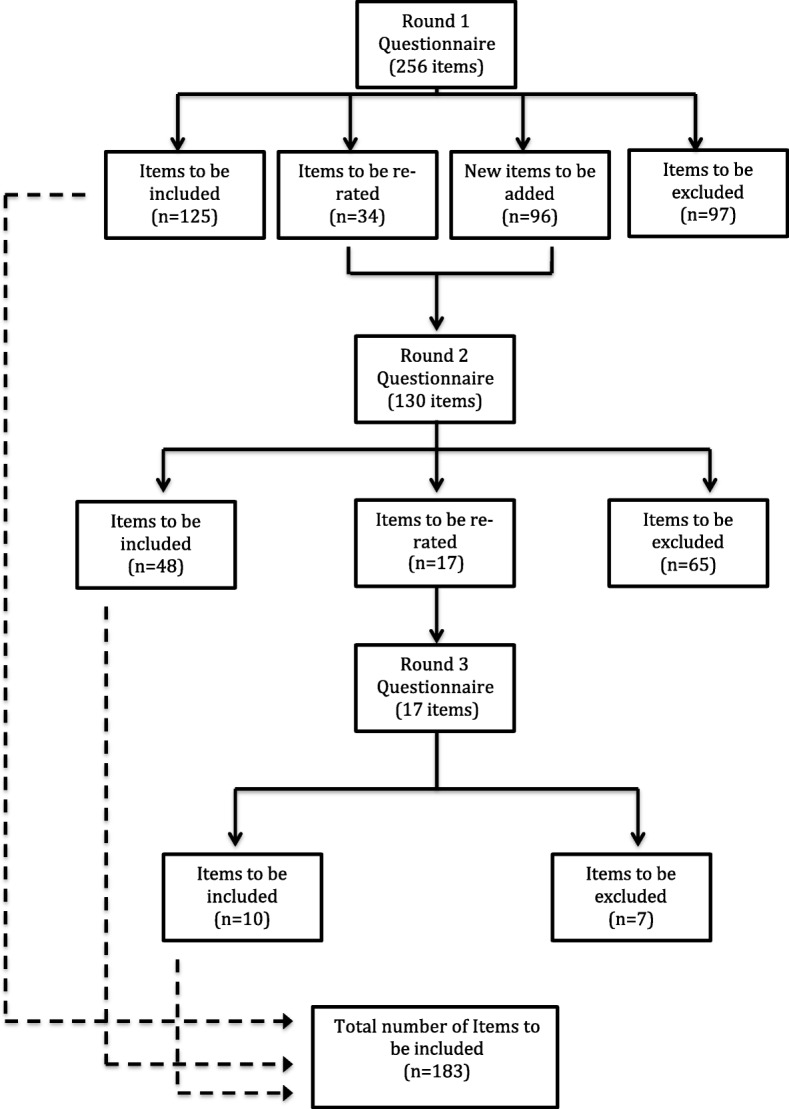
Summary of Item Rating

Three hundred and fifty-two items were rated over the three rounds and a total of 183 were endorsed and 169 rejected. See Fig. 3 for information about the number of items rated, endorsed and rejected. See Additional file 2 for a list of the endorsed and rejected items.

The endorsed items formed the basis of the guidelines document entitled *Depression: Mental Health First Aid Guidelines (Revised 2018)* [23]*,* which will be available from the Mental Health First Aid Australia website (mhfa.com.au). The main topics covered in the guidelines are:

- How do I know if someone is experiencing depression?
- How should I approach someone who may be experiencing depression?
- How can I be supportive?
  - ° Treat the person with respect and dignity
  - ° Offer consistent emotional support and understanding
  - ° Encourage the person to talk to you
  - ° Be a good listener
  - ° Have realistic expectations for the person
  - ° Acknowledge the person’s strengths
  - ° Give the person hope for recovery
  - ° Providing ongoing support
  - ° What does not help?
- What if I experience difficulties when talking to the person?
  - ° Self-care
- Should I encourage the person to seek professional help?
- What about self-help strategies?
- What if the person doesn’t want help?
- What if there is risk of harm to the person or others?

The final draft of the guidelines was provided to participants who completed all three Rounds of the survey for final comments and endorsement. A few minor changes relating to structural composition of the guidelines were made as a result of participant comments.

### Difference between panels

The percentage endorsements for items were strongly positively correlated across the two panels, (*r* = 0.95; t(254) = 48.49; *p* = <.001). However, there were also some differences. As per previous studies (e.g. [12, 24]), items that were endorsed by one panel but rejected by the other, and that received a notably lower rating (±10%) are presented below.

#### *Items rejected by the lived experience panel with a difference of ≥ 10%*

Eighteen items were endorsed by the professional panel but received a lower rating from the lived experience panel:

- Use of diagnostic terms
  - ° The first aider should tell the person that depression is common.
  - ° The first aider should tell the person that depression is an illness.
  - ° If the first aider thinks someone may be depressed, they should approach the person about their concerns.
- Evidence base
  - ° The first aider should tell the person about options for getting evidence-based online or telephone mental health services.
  - ° If the person is interested in self-help strategies, the first aider should provide them with a range of information about evidence-based self-help strategies.
  - ° If the person is interested in self-help strategies, the first aider should encourage the person to use evidence-based strategies.
- Recovery/getting help
  - ° The first aider should let the person know that getting better takes time, but that it will happen.
  - ° The first aider should encourage the person to participate in some activities that once gave them pleasure, e.g. hobbies, sport, religious or cultural activities
  - ° The first aider should continue to involve the person in any activities that they have shared previously.
  - ° The first aider should offer to assist the person to investigate available sources of help.
  - ° The first aider should ask the person if they have tried to get help.
  - ° The first aider should ask the person how much involvement they want the first aider to have with planning for and attending their appointment.
- Distorted thinking
  - ° If the person appears irrational, the first aider should not try to talk the person out of their thoughts or feelings.
  - ° The first aider should not agree with distorted negative thoughts, as these are a symptom of depression.
- Other
  - ° The first aider should ask the person if anyone else knows how they are feeling.
  - ° The first aider should tell the person that they are not to blame for feeling ‘down’.
  - ° The first aider should learn about depression by seeking advice from a mental health professional.
  - ° If the first aider does not feel that they are able to help the person, they should ask someone else to take on the first aider role.

#### *Items rejected by the professional panel with a difference of ≥ 10%*

There were five items that were endorsed by the lived experience panel and received a notably lower rating from the professional panel:

- The first aider should not use a ‘tough-love’ approach to try and make the person better, e.g. the first aider telling the person they will not spend time with them until they get better or get professional help.
- The first aider should offer emotional support and hope of a more positive future in whatever form the depressed person will accept.
- If assisting someone from a cultural background that is different from the first aider’s, the first aider should learn about how depression symptoms may manifest in people from the person’s cultural background.
- If the person does not have the energy or is not able to think clearly enough to investigate available sources of help, the first aider should offer to assist with this.
- If the person refuses to seek or accept professional help, the first aider should ask the person whether they would like the first aider to check in on them.

### Differences between the 2008 and 2018 guidelines

A total of 64 items were endorsed and included in the 2008 guidelines. These endorsed items were included in the 2018 Delphi survey in addition to new items gleaned from the literature search. One hundred and eighty-three items were endorsed and included in the 2018 guidelines. There were 58 items that were endorsed in both the 2008 and 2018 Delphi studies. There were 125 additional items endorsed in the 2018 study. See Additional file 2 for a comparison of item ratings from the 2008 and 2018 studies.

There were some similarities and differences noted between the 2008 and 2018 guidelines. For the 64 survey items that appeared in both the current and the 2008 Delphi, the endorsement ratings were similar. The endorsement rates for survey items in the 2018 study were found to correlate with those in the 2008 study as follows:

- Professional panels - Pearson’s correlation of r = .43 (*t(45)* = 3.20, *p* = .003)
- Lived experience panels – Pearson’s correlation of r = .43 (t(45) = 3.21, *p* = .002).

Note that only endorsed items from the 2008 study were included in the 2018 study, which reduced the range of ratings and is likely to have reduced the correlations.

## Discussion

This research aimed to redevelop guidelines published in 2008 that give advice on how to provide mental health first aid to someone who may be experiencing depression. One hundred and eighty-three items were endorsed by both expert panels and were included in the guidelines. The guidelines will be available to the public on the MHFA Australia website (mhfa.com.au) and they will inform future editions of MHFA Australia courses. They will also be used to develop user-friendly infographics that will be available to the public on the MHFA Australia website.

These guidelines address a variety of topics or situations that a person may encounter when providing mental health first aid to someone who may be experiencing depression. These include recognising the signs of depression in a person, talking with the person about their concerns, how to support the person, what to do if difficulties such as communication problems are encountered, how to encourage help-seeking and what to do if there is risk of harm to the person or others.

### Differences between the two 2018 expert panels

There were a number of items that received a notably different rating score between the two panels. These were categorised into groups – Use of diagnostic terms, Evidence base, Recovery/getting help, Distorted thinking, and Other. Using the qualitative data collected in the Round 1 survey, the reason for the differences between the rating scores of the two panels could be hypothesised. First, there were four items about how the first aider should approach distorted thinking. None of these items reached consensus to be included in the guidelines. However, two items were endorsed by the professional panel, but not by the lived experience panel. The two items were actions that the first aider should **not** do (*The first aider should not agree with distorted negative thoughts, as these are a symptom of depression* and *If the person appears irrational, the first aider should not try to talk the person out of their thoughts or feelings*). The comments suggest that the lived experience panel thought it was appropriate to acknowledge the person’s distorted thinking. Lived experience panel members commented that the first aider needed to have sufficient experience or skills to talk about distorted thoughts in a constructive way. One lived-experience panel member said, “Negative thoughts can be discussed within a conversation but should not become the focus of a conversation.” And another said, “This highly depends on the experience of the first aider, if they do not feel equipped to safely discuss the irrational thoughts then they shouldn’t take it upon themselves to delve deeper as it may reveal/trigger other issues.”

A number of items that implied that the first aider may be labelling or diagnosing the person as having depression were not endorsed by the lived-experience panel, e.g. *The first aider should tell the person that depression is an illness.* The lived experience panel thought that it was important to not label the person as having depression, but rather “…highlight [the] symptoms the [person is] showing…”. Another lived-experience participant said, “[It is] better to discuss symptoms and that they are often associated with depression, and that this might be something to explore, rather than providing a diagnosis.”

### Differences between the 2008 and 2018 guidelines

There were a number of differences noted between the 2008 and 2018 guidelines. The 2018 guidelines included 125 additional items, allowing them to be more nuanced. The complexity of depression is better represented in the re-developed guidelines, for example the item *The first aider should not ignore any signs or symptoms of depression that they have noticed or assume that they will just go away* was endorsed in both 2008 and 2018, but *The first aider should not assume that the person’s symptoms are due to depression* was an additional item in the 2018 re-development, illustrating the complexity in attributing symptoms of mental illness. The re-developed guidelines also allow for a more considered approach to the person when offering help, for example two new items to the 2018 guidelines are:

*The first aider should consider whether they are the best person to approach the person or whether somebody else might be more appropriate.*

*If the first aider thinks someone may be depressed, they should try to spend time with the person and gently bring up their concerns with them, e.g. mention that the person seems down today.*

The mental health first aider role is better defined in the re-developed guidelines. For example, one item that was endorsed in 2008, but not in 2018, was *The first aider needs to let the person with depression know that they will not be abandoned*. The rejection of this item in the 2018 study recognises the limitations and needs of the first aider. One lived-experience participant said, “The first aider may find themselves unable to offer ongoing support due to personal or professional circumstances…The first aider should not feel trapped in a caregiving role.”

The first aid guidance is also more detailed in the 2018 guidelines. For example, the sections on ‘self-help’ and ‘what to do if the person does not want help’ have an additional six and five items, respectively. The additional items encourage the first aider to know more about self-help and help-seeking and respect the person’s ideas about what might be helpful. Although the additional detail may be in some respects helpful, it may also add complexity to the training and this will need careful consideration when updating the course.

Items about first aiders’ knowledge of evidence-based treatments, services or self-help strategies were generally not endorsed. The qualitative data suggested that knowing evidence-based information was outside the role of the first aider. A lived-experience participant said, “Whether treatment is evidence based or how treatment might help or even be undertaken is really beyond the scope of first aid.” Finally, the 2018 guidelines introduced a first aider self-care section.

### Strengths and limitations

Delphi method studies typically use one expert panel, usually professionals with expertise in the area of study [25]. However, multiple expert panels, including consumer and carer participants were used for this Delphi study, mirroring similar recent work in the mental health field [12, 24]. This allows the voice of lived experience to contribute equally to the development of guidelines, which is a strength of this study.

There are a few limitations to this study. Because participants may have been asked to rate survey items that were outside their area of expertise, key actions may have been omitted. Also, participants were not able to discuss their responses with others, which may have led to biases or incorrect assumptions influencing their responses. However, this limitation was ameliorated in that, by eliminating ‘consensus by discussion’, all voices (including quiet or less confident, but equally valid voices) influence the endorsement process just as powerfully. Another limitation is that the professional panel did not include some types of clinicians, such as psychiatrists and primary care physicians. However, as these are not clinical practice guidelines, these experts would have had less relevant expertise than some other professional groups. Finally, by only reviewing the first 50 websites, books and journal articles some first aid actions may have been missed. However, this limitation was minimised because participants could write in missing first aid actions.

## Conclusion

This project used the consensus of consumers, carers and professionals to re-develop the mental health first aid guidelines for depression. This Delphi study ensures that the guidelines that inform the Mental Health First Aid Australia courses and the courses delivered by their international counterparts are current and include the most appropriate helping actions. These updated guidelines are now more detailed, allowing for a more nuanced approach to providing first aid to someone with depression. These guidelines (and the associated infographic) are available on the Mental Health First Aid website, and will be used to update future versions of the Mental Health First Aid Australia course.

## Additional files

Additional file 1: Survey Questionnaire. (PDF 1582 kb)

Additional file 2: Results of Item Rating. (XLSX 51 kb)

## Data Availability

All data generated or analysed during this study are included in this published article as a supplementary file. The datasets analysed during the current study are available from the corresponding author on reasonable request.

## Abbreviation

MHFA: Mental Health First Aid
