# Supplementary material for: Offering mental health first aid to a person with depression: a Delphi study to re-develop the guidelines published in 2008
Source: BMC Psychol. 2019 Jun 21;7:37. doi: 10.1186/s40359-019-0310-3 (PMC6588870; doi:10.1186/s40359-019-0310-3)
Supplement: Supplementary file 1 — Survey Questionnaire. (PDF 1582 kb) [file 40359_2019_310_MOESM1_ESM.pdf]

Updating the Mental Health First Aid Guidelines for Helping someone with Depression

Information about this research

Purpose of this research

The aim of this current research project is to update the mental health first aid guidelines for how a member of the public should give assistance to a person who is experiencing depression. These guidelines are being developed for high income Western countries.

Do you meet the criteria to participate in this study?

You have been invited to participate in this research because you are 18 years or over **AND**:

- Have a lived experience of depression, feel well enough to participate, **AND** are engaged in activities that give you a broader exposure to people's experiences of depression, e.g. you are a member of a consumer advisory or advocacy group, providing peer support to others, etc.

OR

- Have experience in caring for or providing significant day-to-day support to someone with depression **AND** are engaged in activities that give you a broader exposure to people's experiences of depression, e.g. be a member of a carer support group or carer advocacy organisation, etc.

OR

- Are a mental health professional or researcher with at least 2 years experience working in the area of depression.

* 1. Do you meet these criteria?

○ Yes

○ No

How this questionnaire was developed

The statements in this questionnaire were derived from information collected during a search of websites, books, and journal articles on how to provide mental health first aid to somebody with depression.

Some of the statements may seem contradictory or controversial; however, we have included them because they reflect the wide range of people's beliefs about the best ways to provide mental health first aid to someone with depression. It is important to note that we do not necessarily agree with these statements; we have included them because we do not believe that we should decide what the best practice is in this area. Rather, we have invited you to be a member of an expert panel to help develop a set of guidelines that reflect current expert opinion.

You will note that there is a place for you to add comments at the end of each section. This is so you can suggest any additional helping statements you think are important to providing mental health first aid to someone with depression. These statements will then go into the Round 2 survey to be rated by the expert panels.

Definitions used in this survey

Mental health first aid is the help offered to a person developing a mental health problem, experiencing a worsening of an existing mental health problem, or in a mental health crisis. The first aid is given until appropriate professional help is received or until the crisis resolves.

The person refers to the person who the mental health first aider is concerned may be experiencing depression.

The first aider refers to a concerned family member, friend, work colleague or member of the community, who provides help to a person who may be experiencing depression.

Mental health professional refers to a trained health professional who is treating/working with the person. This could be a psychologist, mental health nurse or psychiatrist. It could also be another health professional who has specialist mental health training, e.g. social worker, occupational therapist or GP.

Instructions

Please complete the questionnaire by rating each statement ***according to how important you believe it is for inclusion in the guidelines*** for providing mental health first aid to a person who may be experiencing depression. Please keep in mind that the guidelines will be used by the general public. The statements need to be rated according to their importance for someone ***without a counselling or clinical background***.

This questionnaire should take approximately 45 minutes to complete. You can complete the survey in two or more sittings. Your answers are saved when you click 'Next' at the bottom of a page. This marks your page and you can begin again at a later date on the next page. ***Please be aware that once you have logged on and started responding you must complete the questionnaire on the same computer.***

Updating the Mental Health First Aid Guidelines for Helping someone with Depression

Consent to participate

Consent to participate

It is important for you to know that participation in this study is completely voluntary. You are not under any obligation to participate and you can withdraw at any time.

We would like to thank you for your time and effort, and encourage you to provide us with feedback on this process.

Best wishes,
The Mental Health First Aid Research Team

* 2. I understand that by submitting this survey I am giving my consent to participate in this study.

- ◯ Yes, I understand.
- ◯ I do not consent to participating in this research.

Overview of the study
Section 1: Learning about depression
Section 2: Approaching someone who may be experiencing depression
Section 3: Being supportive
Section 4: Communicating effectively
Section 5: Encouraging help seeking
Section 6: Encouraging self-help strategies
Section 7: What to do if they do not want help
Section 8: Difficulties the first aider may encounter
Section 9: Concerns for safety

Updating the Mental Health First Aid Guidelines for Helping someone with Depression

Information about you

* 3. What is your name? (This allows us to determine who has completed the Round 1 survey and is therefore eligible to participate in Round 2. Your name will be deleted from your data when the project is complete).

* 4. How old are you (in years)?

* 5. What is your gender:

- Female
- Male
- Other
- Do not wish to disclose.

* 6. Please indicate your primary source of expertise, i.e. lived experience, carer or professional.

* 7. In addition to your primary source of expertise, do you also have experience of depression as a (tick all that apply):

- [ ] Person with lived experience of depression
- [ ] Mental health carer or significant support person
- [ ] Mental health professional or researcher
- [ ] No other experience of depression

* 8. Please state the name of the organisation/s you work or volunteer for that make you eligible to participate in this study?

* 9. What is your role within the above organisation/s?

* 10. What country do you live in?

Updating the Mental Health First Aid Guidelines for Helping someone with Depression

Learning about depression

Please rate how important (from 'essential' to 'should not be included') you think it is that each statement be included in the guidelines.

Please keep our definitions in mind when responding.

* 11. The first aider should know the following:

	Essential	Important	Depends/Don't know	Unimportant	Should not be included
Signs and symptoms of depression	○	○	○	○	○
Risk factors for depression.	○	○	○	○	○

* 12. The first aider should be aware that the experience of depression can vary in severity, e.g. from feeling irritable to feeling suicidal.

- Essential
- Important
- Don't know/Depends
- Unimportant
- Should not be included

* 13. The first aider should be aware that each individual is different and not everyone who is experiencing depression will show the typical signs or symptoms of depression.

- Essential
- Important
- Don't know/Depends
- Unimportant
- Should not be included

* 14. The first aider should be aware that some people who have recovered from depression may have a relapse of their symptoms.

- Essential
- Important
- Don't know/Depends
- Unimportant
- Should not be included

* 15. Even if the person has had a previous episode of depression, the first aider should not assume they will know how to manage the current episode.

- Essential
- Important
- Don't know/Depends
- Unimportant
- Should not be included

* 16. The first aider should learn more about depression by:

	Essential	Important	Depends/Don't know	Unimportant	Should not be included
Reading about or listening to other people's experiences of depression	○	○	○	○	○
Reading reputable information websites or books	○	○	○	○	○
Seeking advice from other people who know someone with depression	○	○	○	○	○
Seeking advice from people who have experienced depression	○	○	○	○	○
Seeking advice from a mental health professional	○	○	○	○	○
Seeking advice from an online or telephone help service.	○	○	○	○	○

17. Please provide any additional items or comments related to this section.

Updating the Mental Health First Aid Guidelines for Helping someone with Depression

Approaching someone who may be experiencing depression

Please rate how important (from ‘essential’ to ‘should not be included’) you think it is that each statement be included in the guidelines.

Please keep our definitions in mind when responding.

If the first aider notices signs or symptoms of depression

* 18. The first aider should not ignore any signs or symptoms of depression that they have noticed or assume that they will just go away.

- Essential
- Important
- Don't know/Depends
- Unimportant
- Should not be included

* 19. The first aider should not assume that any signs or symptoms they have noticed means that the person is experiencing depression.

- Essential
- Important
- Don't know/Depends
- Unimportant
- Should not be included

* 20. The first aider should know that many people who experience depression may also be affected by other mental health problems like anxiety or substance use problems.

- Essential
- Important
- Don't know/Depends
- Unimportant
- Should not be included

* 21. The first aider should not lie or make excuses for the person’s behaviour as this may delay them getting assistance.

- Essential
- Important
- Don't know/Depends
- Unimportant
- Should not be included

* 22. If the first aider thinks someone may be depressed, they should approach the person about their concerns.

- Essential
- Important
- Don't know/Depends
- Unimportant
- Should not be included

Preparing for the conversation

* 23. The first aider should choose a suitable time when both the first aider and the person have the time to talk.

- Essential
- Important
- Don't know/Depends
- Unimportant
- Should not be included

* 24. The first aider should choose a place to talk that is private.

- Essential
- Important
- Don't know/Depends
- Unimportant
- Should not be included

* 25. The first aider should choose a place to talk where both they and the person feel comfortable.

- Essential
- Important
- Don't know/Depends
- Unimportant
- Should not be included

26. Please provide any additional items or comments related to this section.

Updating the Mental Health First Aid Guidelines for Helping someone with Depression

Approaching someone who may be experiencing depression (cont)

Please rate how important (from ‘essential’ to ‘should not be included’) you think it is that each statement be included in the guidelines.

Please keep our definitions in mind when responding.

Starting a conversation

* 27. If the first aider is worried about someone who may be depressed, they should:

	Essential	Important	Depends/Don't know	Unimportant	Should not be included
Tell the person that they care and want to help.	○	○	○	○	○
Tell the person about the specific changes that they have noticed in a supportive and sensitive manner.	○	○	○	○	○
Let the person choose when to open up.	○	○	○	○	○

* 28. If the person does not initiate a conversation about how they are feeling, the first aider should take a direct approach and raise the topic with them.

- ○ Essential
- ○ Important
- ○ Don't know/Depends
- ○ Unimportant
- ○ Should not be included

* 29. If the person says that they are feeling sad or down, the first aider should ask them how long they have been feeling that way.

- Essential
- Important
- Don't know/Depends
- Unimportant
- Should not be included

* 30. The first aider should try to explore the person’s beliefs about depression with them.

- Essential
- Important
- Don't know/Depends
- Unimportant
- Should not be included

* 31. The first aider should respect the person's interpretation of their signs and symptoms.

- Essential
- Important
- Don't know/Depends
- Unimportant
- Should not be included

* 32. The first aider should not assume that the person knows nothing about depression.

- Essential
- Important
- Don't know/Depends
- Unimportant
- Should not be included

* 33. The first aider should suggest to the person that depression might be the problem.

- Essential
- Important
- Don't know/Depends
- Unimportant
- Should not be included

* 34. The first aider should tell the person that depression is common.

- Essential
- Important
- Don't know/Depends
- Unimportant
- Should not be included

* 35. The first aider should discuss their own understanding of the signs and symptoms of depression with the person.

- Essential
- Important
- Don't know/Depends
- Unimportant
- Should not be included

36. Please provide any additional items or comments related to this section.

Updating the Mental Health First Aid Guidelines for Helping someone with Depression

Approaching someone who may be experiencing depression (cont)

Please rate how important (from 'essential' to 'should not be included') you think it is that each statement be included in the guidelines.

Please keep our definitions in mind when responding.

Giving the person information

* 37. The first aider should ask the person if they would like some information about depression.

- Essential
- Important
- Don't know/Depends
- Unimportant
- Should not be included

* 38. The first aider should know sources of good quality information for the person.

- Essential
- Important
- Don't know/Depends
- Unimportant
- Should not be included

* 39. The first aider should provide the person with good quality information about depression (e.g. pamphlets or website) and encourage them to use it.

- Essential
- Important
- Don't know/Depends
- Unimportant
- Should not be included

* 40. If the first aider gives the person information about depression, they should try to ensure that the resources are accurate and appropriate to the person’s situation.

- Essential
- Important
- Don't know/Depends
- Unimportant
- Should not be included

When talking to the person

* 41. When approaching the person, the first aider should not directly tell them that they need help.

- Essential
- Important
- Don't know/Depends
- Unimportant
- Should not be included

* 42. If the person doesn't feel comfortable talking to the first aider, they should encourage the person to discuss how they are feeling with someone else.

- Essential
- Important
- Don't know/Depends
- Unimportant
- Should not be included

* 43. The first aider should prepare for the full range of reactions (e.g. relief, indifference, anger) when they approach the person about their depression.

- Essential
- Important
- Don't know/Depends
- Unimportant
- Should not be included

44. Please provide any additional items or comments related to this section.

Updating the Mental Health First Aid Guidelines for Helping someone with Depression

Approaching someone who may be experiencing depression (cont)

Please rate how important (from 'essential' to 'should not be included') you think it is that each statement be included in the guidelines.

Please keep our definitions in mind when responding.

When talking to the person (cont)

* 45. The first aider should know that each person's situation and needs are unique.

- Essential
- Important
- Don't know/Depends
- Unimportant
- Should not be included

* 46. The first aider should respect the person's autonomy while considering the extent to which the person is able to make decisions for themselves and whether the person is at risk of harming themselves or others.

- Essential
- Important
- Don't know/Depends
- Unimportant
- Should not be included

* 47. The first aider should keep private any discussions they have with the person unless they are concerned that the person may harm themselves or others.

- Essential
- Important
- Don't know/Depends
- Unimportant
- Should not be included

* 48. The first aider should let the person know in advance that they will intervene and seek professional help for the person if they ever believe the person's life may be in danger.

- Essential
- Important
- Don't know/Depends
- Unimportant
- Should not be included

* 49. The first aider should use the same terminology that the person uses when discussing their experience, e.g. if they say they are "moody" or "down", use these terms rather than "depressed", or vice versa.

- Essential
- Important
- Don't know/Depends
- Unimportant
- Should not be included

50. Please provide any additional items or comments related to this section.

Updating the Mental Health First Aid Guidelines for Helping someone with Depression

Being supportive

Please rate how important (from ‘essential’ to ‘should not be included’) you think it is that each statement be included in the guidelines.

Please keep our definitions in mind when responding.

Understanding depression

* 51. The first aider should know that those who haven’t experienced depression cannot fully understand what it is like for those who have.

- Essential
- Important
- Don't know/Depends
- Unimportant
- Should not be included

* 52. The first aider should tell the person that they are not to blame for feeling 'down'.

- Essential
- Important
- Don't know/Depends
- Unimportant
- Should not be included

* 53. The first aider should let the person know that they are not weak or a failure because they have depression, and that they don't think less of them as a person.

- Essential
- Important
- Don't know/Depends
- Unimportant
- Should not be included

* 54. The first aider should know that depression is a medical illness and it is not the person's fault that they are experiencing depression.

- Essential
- Important
- Don't know/Depends
- Unimportant
- Should not be included

* 55. The first aider should tell the person that depression is an illness.

- Essential
- Important
- Don't know/Depends
- Unimportant
- Should not be included

* 56. The first aider should know that the person is experiencing depression as a set of feelings about themselves, others and the world. Therefore, the first aider should not imply that the person simply has a medical illness, as this may invalidate their experiences.

- Essential
- Important
- Don't know/Depends
- Unimportant
- Should not be included

If the first aider is affected by the conversation

* 57. The first aider should try to see any irritable or unpleasant behaviours as part of the illness and not take these personally.

- Essential
- Important
- Don't know/Depends
- Unimportant
- Should not be included

* 58. If the first aider feels frustrated, they should let the person know they are frustrated with the person's illness, not with them.

- Essential
- Important
- Don't know/Depends
- Unimportant
- Should not be included

* 59. If the person's behaviour is having a negative effect on the first aider, they should try not to show the person how they feel.

- Essential
- Important
- Don't know/Depends
- Unimportant
- Should not be included

* 60. If the person’s behaviour is having a negative effect on the first aider, they should give the person honest feedback about this.

- Essential
- Important
- Don't know/Depends
- Unimportant
- Should not be included

61. Please provide any additional items or comments related to this section.

Updating the Mental Health First Aid Guidelines for Helping someone with Depression

Being supportive (cont)

Please rate how important (from ‘essential’ to ‘should not be included’) you think it is that each statement be included in the guidelines.

Please keep our definitions in mind when responding.

Offering support

* 62. The first aider should know that a depressed person may be overwhelmed by irrational fears.

- Essential
- Important
- Don't know/Depends
- Unimportant
- Should not be included

* 63. The first aider should know that the person may become obsessive in talking about their feelings, making them seem self-absorbed.

- ◯ Essential
- ◯ Important
- ◯ Don't know/Depends
- ◯ Unimportant
- ◯ Should not be included

* 64. The first aider should be patient, persistent and encouraging when supporting someone with depression.

- ◯ Essential
- ◯ Important
- ◯ Don't know/Depends
- ◯ Unimportant
- ◯ Should not be included

* 65. The first aider should know that the person genuinely needs additional support and understanding.

- ◯ Essential
- ◯ Important
- ◯ Don't know/Depends
- ◯ Unimportant
- ◯ Should not be included

* 66. The first aider should offer the person kindness and attention, even if it is not reciprocated.

- ◯ Essential
- ◯ Important
- ◯ Don't know/Depends
- ◯ Unimportant
- ◯ Should not be included

* 67. The first aider should be aware that their support is likely to be having a positive impact, even if it does not feel this way.

- Essential
- Important
- Don't know/Depends
- Unimportant
- Should not be included

* 68. The first aider should let the person know that they will not abandon them.

- Essential
- Important
- Don't know/Depends
- Unimportant
- Should not be included

* 69. The first aider should not use a 'tough-love' approach to try and make the person better, e.g. the first aider telling the person they will not spend time with them until they get better or get professional help.

- Essential
- Important
- Don't know/Depends
- Unimportant
- Should not be included

* 70. The first aider should be consistent and predictable in their interactions with the person.

- Essential
- Important
- Don't know/Depends
- Unimportant
- Should not be included

71. Please provide any additional items or comments related to this section.

Being supportive (cont)

Please rate how important (from ‘essential’ to ‘should not be included’) you think it is that each statement be included in the guidelines.

Please keep our definitions in mind when responding.

Offering support (cont)

* 72. If the person judges themselves too harshly, the first aider should remind the person of their strengths.

- Essential
- Important
- Don't know/Depends
- Unimportant
- Should not be included

* 73. The first aider should acknowledge any efforts the person is making to get better.

- Essential
- Important
- Don't know/Depends
- Unimportant
- Should not be included

* 74. The first aider should tell the person that although their experience is very personal and painful, most of their symptoms are shared by other people with depression.

- Essential
- Important
- Don't know/Depends
- Unimportant
- Should not be included

* 75. The first aider should tell the person that they are trying to imagine how they feel.

- Essential
- Important
- Don't know/Depends
- Unimportant
- Should not be included

* 76. The first aider should complain a little about their life to the person to help the person feel less alone.

- Essential
- Important
- Don't know/Depends
- Unimportant
- Should not be included

* 77. The first aider should know that often just taking the time to talk to or be with the person lets them know that someone cares.

- Essential
- Important
- Don't know/Depends
- Unimportant
- Should not be included

* 78. If appropriate to the relationship, the first aider should offer the person some form of physical contact, such as a hug.

- Essential
- Important
- Don't know/Depends
- Unimportant
- Should not be included

* 79. If possible, the first aider should connect the person with someone who has recovered from depression.

- ◯ Essential
- ◯ Important
- ◯ Don't know/Depends
- ◯ Unimportant
- ◯ Should not be included

80. Please provide any additional items or comments related to this section.

[ ]

Updating the Mental Health First Aid Guidelines for Helping someone with Depression

Being supportive (cont)

Please rate how important (from ‘essential’ to ‘should not be included’) you think it is that each statement be included in the guidelines.

Please keep our definitions in mind when responding.

Offering assistance

* 81. The first aider should ask the person what practical assistance they need.

- ◯ Essential
- ◯ Important
- ◯ Don't know/Depends
- ◯ Unimportant
- ◯ Should not be included

* 82. The first aider should ask the person if they would like any practical assistance with tasks, but should be careful not to take over or encourage dependency.

- ◯ Essential
- ◯ Important
- ◯ Don't know/Depends
- ◯ Unimportant
- ◯ Should not be included

* 83. The first aider should make any decision that they can for the person.

- Essential
- Important
- Don't know/Depends
- Unimportant
- Should not be included

* 84. If the first aider is particularly concerned about the person, they should organise to have close friends or family members to make contact with the person each day, e.g. to provide practical help, have a coffee, simply to say hello.

- Essential
- Important
- Don't know/Depends
- Unimportant
- Should not be included

* 85. If the person lives alone and finds this difficult, the first aider should stay with the person or try to arrange for several friends to take turns staying with the person.

- Essential
- Important
- Don't know/Depends
- Unimportant
- Should not be included

* 86. The first aider should let the person know that stress is a risk factor for depression and encourage them to find ways to reduce stress in their life.

- Essential
- Important
- Don't know/Depends
- Unimportant
- Should not be included

* 87. The first aider should offer to help the person to find strategies to reduce stress in their life.

- ○ Essential
- ○ Important
- ○ Don't know/Depends
- ○ Unimportant
- ○ Should not be included

* 88. The first aider should help the person to focus on positive things in their life, e.g. fill a box with anything that reminds them of what's good in their life such as favourite photographs or letters; keep a journal of positives.

- ○ Essential
- ○ Important
- ○ Don't know/Depends
- ○ Unimportant
- ○ Should not be included

89. Please provide any additional items or comments related to this section.

Updating the Mental Health First Aid Guidelines for Helping someone with Depression

Being supportive (cont)

Please rate how important (from ‘essential’ to ‘should not be included’) you think it is that each statement be included in the guidelines.

Please keep our definitions in mind when responding.

Offering assistance (cont)

* 90. The first aider should encourage the person to lead a healthy lifestyle by being an example to the person, e.g. maintaining a positive outlook, eating better, exercising, etc.

- Essential
- Important
- Don't know/Depends
- Unimportant
- Should not be included

* 91. The first aider should try to make the person laugh.

- Essential
- Important
- Don't know/Depends
- Unimportant
- Should not be included

* 92. The first aider should try to lift the person’s mood or cheer them up.

- Essential
- Important
- Don't know/Depends
- Unimportant
- Should not be included

* 93. The first aider should encourage the person to participate in some activities that once gave them pleasure, e.g. hobbies, sport, religious or cultural activities.

- Essential
- Important
- Don't know/Depends
- Unimportant
- Should not be included

* 94. The first aider should try to provide a change of scenery for the person, e.g. go for a walk in a different area.

- Essential
- Important
- Don't know/Depends
- Unimportant
- Should not be included

* 95. The first aider should be clear and consistent in what support they can and cannot offer to the person.

- Essential
- Important
- Don't know/Depends
- Unimportant
- Should not be included

* 96. The first aider should not make promises they cannot keep.

- Essential
- Important
- Don't know/Depends
- Unimportant
- Should not be included

* 97. The first aider should ask the person whether what they are doing is helpful, and what else they could do to help.

- Essential
- Important
- Don't know/Depends
- Unimportant
- Should not be included

98. Please provide any additional items or comments related to this section.

Being supportive (cont)

Please rate how important (from 'essential' to 'should not be included') you think it is that each statement be included in the guidelines.

Please keep our definitions in mind when responding.

Expectations

* 99. The first aider should accept the person as they are and have realistic expectations for them.

- Essential
- Important
- Don't know/Depends
- Unimportant
- Should not be included

* 100. The first aider should know that everyday activities like cleaning the house, paying bills, or feeding the dog may seem overwhelming to the person.

- Essential
- Important
- Don't know/Depends
- Unimportant
- Should not be included

* 101. The first aider should acknowledge that the person is not 'faking', 'lazy', 'weak' or 'selfish'.

- Essential
- Important
- Don't know/Depends
- Unimportant
- Should not be included

* 102. The first aider should continue to involve the person in any activities that they have shared previously.

- ◯ Essential
- ◯ Important
- ◯ Don't know/Depends
- ◯ Unimportant
- ◯ Should not be included

* 103. The first aider should not push the person to do activities that the person feels are too much for them.

- ◯ Essential
- ◯ Important
- ◯ Don't know/Depends
- ◯ Unimportant
- ◯ Should not be included

* 104. The first aider should offer to help the person modify their day-to-day activities as needed.

- ◯ Essential
- ◯ Important
- ◯ Don't know/Depends
- ◯ Unimportant
- ◯ Should not be included

* 105. The first aider should provide the same support that they would if the person had a physical illness, e.g. sending a get well card or flowers.

- ◯ Essential
- ◯ Important
- ◯ Don't know/Depends
- ◯ Unimportant
- ◯ Should not be included

106. Please provide any additional items or comments related to this section.

Being supportive (cont)

Please rate how important (from 'essential' to 'should not be included') you think it is that each statement be included in the guidelines.

Please keep our definitions in mind when responding.

Messages about recovery

* 107. The first aider should consider telling the person that:

	Essential	Important	Depends/Don't know	Unimportant	Should not be included
They are not alone in this.	◯	◯	◯	◯	◯
The first aider is there for the person.	◯	◯	◯	◯	◯
Although the first aider may not be able to understand exactly how the person feels, they care and want to help.	◯	◯	◯	◯	◯
They are important to the first aider and their life is important.	◯	◯	◯	◯	◯
They may not believe it now, but the way they are feeling will improve.	◯	◯	◯	◯	◯
With time and treatment the person will feel better.	◯	◯	◯	◯	◯

* 108. The first aider should offer emotional support and hope of a more positive future in whatever form the depressed person will accept.

◯ Essential

◯ Important

◯ Don't know/Depends

◯ Unimportant

◯ Should not be included

* 109. The first aider should give the person the message that they believe that the person can resolve their problems themselves.

- Essential
- Important
- Don't know/Depends
- Unimportant
- Should not be included

110. Please provide any additional items or comments related to this section.

Updating the Mental Health First Aid Guidelines for Helping someone with Depression

Being supportive (cont)

Please rate how important (from ‘essential’ to ‘should not be included’) you think it is that each statement be included in the guidelines.

Please keep our definitions in mind when responding.

What not to do or say

* 111. The first aider should know that there is no point in just telling the person to get better.

- Essential
- Important
- Don't know/Depends
- Unimportant
- Should not be included

* 112. The first aider should know that the person can't 'snap out of it' or 'get over it'.

- Essential
- Important
- Don't know/Depends
- Unimportant
- Should not be included

* 113. The first aider should not tell the person that they just need to stay busy or get out more.

- Essential
- Important
- Don't know/Depends
- Unimportant
- Should not be included

* 114. The first aider should not trivialise the person's experiences by telling them to "put a smile on their face," to "get their act together," or to "lighten up".

- Essential
- Important
- Don't know/Depends
- Unimportant
- Should not be included

* 115. The first aider should avoid using the words "I know how you feel" or "I understand".

- Essential
- Important
- Don't know/Depends
- Unimportant
- Should not be included

* 116. The person should avoid saying the following:

	Essential	Important	Depends/Don't know	Unimportant	Should not be included
It's all in your head					
I can't do anything about your situation.					

* 117. Even if the first aider feels that the person is unpleasant to be around, they should not tell them so.

- Essential
- Important
- Don't know/Depends
- Unimportant
- Should not be included

118. Please provide any additional items or comments related to this section.

Updating the Mental Health First Aid Guidelines for Helping someone with Depression

Being supportive (cont)

Please rate how important (from 'essential' to 'should not be included') you think it is that each statement be included in the guidelines.

Please keep our definitions in mind when responding.

What not to do or say (cont)

* 119. The first aider should not adopt an over-involved or over-protective attitude towards the person.

- Essential
- Important
- Don't know/Depends
- Unimportant
- Should not be included

* 120. The first aider should not speak to the person with a patronising tone of voice and should not use overly-compassionate looks of concern.

- Essential
- Important
- Don't know/Depends
- Unimportant
- Should not be included

* 121. The first aider should not nag the person to get them to do what they normally would.

- Essential
- Important
- Don't know/Depends
- Unimportant
- Should not be included

* 122. The first aider should not belittle or dismiss the person's feelings by attempting to say something positive like, "You don't seem that bad to me."

- Essential
- Important
- Don't know/Depends
- Unimportant
- Should not be included

* 123. The first aider should avoid trying to cheer the person up.

- Essential
- Important
- Don't know/Depends
- Unimportant
- Should not be included

* 124. The first aider should not try to distract the person from their depression by pointing out positive things, e.g. lovely weather.

- Essential
- Important
- Don't know/Depends
- Unimportant
- Should not be included

* 125. The first aider should resist the urge to try to cure the person’s depression.

- Essential
- Important
- Don't know/Depends
- Unimportant
- Should not be included

* 126. The first aider should resist the urge to try to come up with solutions to the person's problems.

- Essential
- Important
- Don't know/Depends
- Unimportant
- Should not be included

* 127. The first aider should not ask the person why they are depressed.

- Essential
- Important
- Don't know/Depends
- Unimportant
- Should not be included

* 128. The first aider should not suggest to the person that they should use alcohol or other drugs to feel better.

- Essential
- Important
- Don't know/Depends
- Unimportant
- Should not be included

129. Please provide any additional items or comments related to this section.

Communicating effectively

Please rate how important (from 'essential' to 'should not be included') you think it is that each statement be included in the guidelines.

Please keep our definitions in mind when responding.

Talking to the person

* 130. It is more important for the first aider to be genuinely caring than for them to say all the 'right things'.

- Essential
- Important
- Don't know/Depends
- Unimportant
- Should not be included

* 131. The first aider should encourage the person to talk about their thoughts, feelings, symptoms and any other problems they are experiencing.

- Essential
- Important
- Don't know/Depends
- Unimportant
- Should not be included

* 132. The first aider should know that some people may find it difficult to discuss their thoughts and feelings openly. If this is the case, the first aider should suggest going for a walk together, as this may make it easier for the person to talk.

- Essential
- Important
- Don't know/Depends
- Unimportant
- Should not be included

* 133. The first aider should not put pressure on the person to talk right away.

- Essential
- Important
- Don't know/Depends
- Unimportant
- Should not be included

* 134. If the person does not have the energy or inclination to discuss how they are feeling, the first aider should not put pressure on them to do so.

- Essential
- Important
- Don't know/Depends
- Unimportant
- Should not be included

* 135. The first aider should not be hostile or sarcastic when the person is struggling to respond, but rather accept these responses as the best the person has to offer at that time.

- Essential
- Important
- Don't know/Depends
- Unimportant
- Should not be included

* 136. If the person does not want to talk, the first aider should let them know that they are available to talk when they are ready.

- Essential
- Important
- Don't know/Depends
- Unimportant
- Should not be included

137. Please provide any additional items or comments related to this section.

Communicating effectively (cont)

Please rate how important (from ‘essential’ to ‘should not be included’) you think it is that each statement be included in the guidelines.

Please keep our definitions in mind when responding.

Talking to the person (cont)

* 138. The first aider should tell the person how they are being affected by the person's depression.

- Essential
- Important
- Don't know/Depends
- Unimportant
- Should not be included

* 139. If the first aider has had personal experiences with depression, they should share these with the person.

- Essential
- Important
- Don't know/Depends
- Unimportant
- Should not be included

* 140. If the first aider has had personal experiences with depression, they should avoid sharing these with the person as it may not be helpful, e.g. the person may feel their experiences are being minimised.

- Essential
- Important
- Don't know/Depends
- Unimportant
- Should not be included

* 141. The first aider should ask open-ended questions to give the person an opportunity to say what they want to.

- Essential
- Important
- Don't know/Depends
- Unimportant
- Should not be included

* 142. The first aider should ask specific rather than general questions, e.g. rather than asking "How are you?" they should ask, "How are you today compared to yesterday?"

- Essential
- Important
- Don't know/Depends
- Unimportant
- Should not be included

* 143. The first aider should use closed questions when the person is unable to respond to open questions.

- Essential
- Important
- Don't know/Depends
- Unimportant
- Should not be included

* 144. The first aider should engage the person in short conversations rather than long talks.

- Essential
- Important
- Don't know/Depends
- Unimportant
- Should not be included

* 145. The first aider should ask the person how the are feeling on a regular basis.

- Essential
- Important
- Don't know/Depends
- Unimportant
- Should not be included

* 146. The first aider should ask the person how they are feeling on a scale of 1 to 10 where 1 is suicidal and 10 is feeling 100% normal.

- Essential
- Important
- Don't know/Depends
- Unimportant
- Should not be included

* 147. The first aider should listen carefully to the person even if what they are saying is obviously not true or is misguided.

- Essential
- Important
- Don't know/Depends
- Unimportant
- Should not be included

148. Please provide any additional items or comments related to this section.

Updating the Mental Health First Aid Guidelines for Helping someone with Depression

Communicating effectively (cont)

Please rate how important (from 'essential' to 'should not be included') you think it is that each statement be included in the guidelines.

Please keep our definitions in mind when responding.

Listing to the person

* 149. The first aider should listen to the person without expressing judgment.

- Essential
- Important
- Don't know/Depends
- Unimportant
- Should not be included

* 150. The first aider should know that the key attitudes involved in non-judgmental listening are acceptance, genuineness and empathy.

- Essential
- Important
- Don't know/Depends
- Unimportant
- Should not be included

* 151. The first aider should respect the person's feelings, personal values and experiences as valid, even if they are different from their own, or they disagree with them.

- Essential
- Important
- Don't know/Depends
- Unimportant
- Should not be included

* 152. The first aider should be aware that the person themselves may hold stigmatising attitudes towards mental illness and should model acceptance.

- Essential
- Important
- Don't know/Depends
- Unimportant
- Should not be included

* 153. The first aider should convey genuineness to the person by using body language that matches their verbal communication, e.g. telling the person you accept and respect their feelings, while maintaining an open posture and appropriate eye contact.

- Essential
- Important
- Don't know/Depends
- Unimportant
- Should not be included

* 154. The first aider should demonstrate empathy by showing the person that they are truly heard and understood, e.g. saying, "What you are going through must be difficult."

- Essential
- Important
- Don't know/Depends
- Unimportant
- Should not be included

* 155. The first aider should adopt an attitude of acceptance of the person by:

	Essential	Important	Depends/Don't know	Unimportant	Should not be included
Withholding any and all judgments that you have made about the person or their circumstances, e.g. if the first aider feels the person is being lazy, they should not express this.	○	○	○	○	○
Choosing words carefully so as to avoid causing offence, e.g. not applying any labels to the person that they may find stigmatising, such as 'mentally ill'.	○	○	○	○	○

156. Please provide any additional items or comments related to this section.

Communicating effectively (cont)

Please rate how important (from ‘essential’ to ‘should not be included’) you think it is that each statement be included in the guidelines.

Please keep our definitions in mind when responding.

Listening to the person (cont)

* 157. The first aider should use the following verbal skills to show they are listening:

	Essential	Important	Depends/Don't know	Unimportant	Should not be included
Asking questions that show that they genuinely care and want to understand what the person is saying.	○	○	○	○	○
Checking their understanding by restating what the person has said and summarising facts and feelings.	○	○	○	○	○
Listening not only to what the person says, but how they say it, e.g. their tone of voice.	○	○	○	○	○
Using minimal prompts, such as “I see” and “Mmmm” when necessary to keep the conversation with the person going.	○	○	○	○	○
Being patient, even when the person may not be communicating well, may be repetitive or may be speaking slower and less clearly than usual.	○	○	○	○	○
Not being critical or expressing their frustration at the person for not being able to communicate well.	○	○	○	○	○

	Essential	Important	Depends/Don't know	Unimportant	Should not be included
Avoiding giving unhelpful advice such as "Pull yourself together" or "Cheer up." If this was possible the person would do it.	○	○	○	○	○
Not interrupting the person when they are speaking, especially to share their own opinions or experiences.	○	○	○	○	○
Avoiding confrontation, unless necessary to prevent the person from committing harmful or dangerous acts.	○	○	○	○	○

* 158. The first aider should know that pauses and silences are okay and that while they may feel uncomfortable, the person may need time to think or find the right words.

○ Essential

○ Important

○ Don't know/Depends

○ Unimportant

○ Should not be included

159. Please provide any additional items or comments related to this section.

Updating the Mental Health First Aid Guidelines for Helping someone with Depression

Communicating effectively (cont)

Please rate how important (from 'essential' to 'should not be included') you think it is that each statement be included in the guidelines.

Please keep our definitions in mind when responding.

Non-verbal communication

* 160. The first aider should use the following non-verbal skills to reinforce their non-judgmental communication:

	Essential	Important	Depends/Don't know	Unimportant	Should not be included
Paying close attention to what the person says.	◯	◯	◯	◯	◯
Maintaining comfortable eye contact by using the level of eye contact that the person seems most comfortable with.	◯	◯	◯	◯	◯
Being aware of the person's body language, as this can provide clues as to how they are feeling or how comfortable they feel about talking with the first aider.	◯	◯	◯	◯	◯
Noticing how much personal space the person feels comfortable with and respecting that.	◯	◯	◯	◯	◯
Maintaining an open body position, e.g. not crossing arms, as this may appear defensive.	◯	◯	◯	◯	◯
If it is safe, sitting down, even if the person is standing. This may seem less threatening.	◯	◯	◯	◯	◯
Sitting alongside the person and angled towards them, rather than directly opposite them.	◯	◯	◯	◯	◯
Avoiding distracting gestures (e.g. fidgeting with a pen, glancing at other things or tapping your feet or fingers), as these could be interpreted as a lack of interest.	◯	◯	◯	◯	◯

* 161. Set aside any negative beliefs and reactions in order to focus on the needs of the person they are helping.

- Essential
- Important
- Don't know/Depends
- Unimportant
- Should not be included

* 162. The first aider should know that helping someone who is depressed may evoke an unexpected emotional response in the first aider.

- Essential
- Important
- Don't know/Depends
- Unimportant
- Should not be included

163. Please provide any additional items or comments related to this section.

Updating the Mental Health First Aid Guidelines for Helping someone with Depression

Communicating effectively (cont)

Please rate how important (from 'essential' to 'should not be included') you think it is that each statement be included in the guidelines.

Please keep our definitions in mind when responding.

Cultural considerations when communicating with the person

* 164. If assisting someone from a cultural background that is different from the first aider's, the first aider should be willing to adjust their verbal and non-verbal behaviours, e.g. the person may be comfortable with a different level of eye contact, or may be used to more personal space.

- Essential
- Important
- Don't know/Depends
- Unimportant
- Should not be included

* 165. If cultural differences are interfering with the first aider's ability to help the person, they should:

	Essential	Important	Depends/Don't know	Unimportant	Should not be included
Explore the person's experiences, values or belief systems with them.	○	○	○	○	○
Discuss with the person what is culturally appropriate and realistic for them.	○	○	○	○	○
Seek advice from someone from the person's cultural background.	○	○	○	○	○

166. Please provide any additional items or comments related to this section.

Updating the Mental Health First Aid Guidelines for Helping someone with Depression

Help-seeking

Please rate how important (from ‘essential’ to ‘should not be included’) you think it is that each statement be included in the guidelines.

Please keep our definitions in mind when responding.

When to encourage help-seeking

* 167. The first aider should not assume the person's depression will just go away.

- Essential
- Important
- Don't know/Depends
- Unimportant
- Should not be included

* 168. The first aider should know that treating depression early is important to getting the best outcomes.

- Essential
- Important
- Don't know/Depends
- Unimportant
- Should not be included

* 169. The first aider should be able to recognise when to encourage the person to seek professional help.

- Essential
- Important
- Don't know/Depends
- Unimportant
- Should not be included

* 170. The first aider should ask the person if they need professional help to manage how they are feeling.

- Essential
- Important
- Don't know/Depends
- Unimportant
- Should not be included

* 171. The first aider should encourage the person to get professional help.

- Essential
- Important
- Don't know/Depends
- Unimportant
- Should not be included

* 172. The first aider should encourage the person to get professional help***as early as possible***.

- Essential
- Important
- Don't know/Depends
- Unimportant
- Should not be included

* 173. The first aider should not encourage professional help seeking unless the person asks about it.

- Essential
- Important
- Don't know/Depends
- Unimportant
- Should not be included

* 174. The first aider should ask the person if they have tried to get help.

- Essential
- Important
- Don't know/Depends
- Unimportant
- Should not be included

* 175. The first aider should discuss the options that the person has for seeking help and should encourage them to use these options.

- Essential
- Important
- Don't know/Depends
- Unimportant
- Should not be included

* 176. The first aider should mention to the person that it is important to see a medical professional as other medical conditions, such as thyroid disease, can mimic the symptoms of depression.

- Essential
- Important
- Don't know/Depends
- Unimportant
- Should not be included

177. Please provide any additional items or comments related to this section.

Updating the Mental Health First Aid Guidelines for Helping someone with Depression

Help-seeking (cont)

Please rate how important (from 'essential' to 'should not be included') you think it is that each statement be included in the guidelines.

Please keep our definitions in mind when responding.

How to assist the person with help-seeking

* 178. The first aider should help the person find a mental health professional.

- Essential
- Important
- Don't know/Depends
- Unimportant
- Should not be included

* 179. The first aider should offer to assist the person to investigate available sources of help.

- Essential
- Important
- Don't know/Depends
- Unimportant
- Should not be included

* 180. If the person does not know where to get help, the first aider should offer to help them seek assistance.

- Essential
- Important
- Don't know/Depends
- Unimportant
- Should not be included

* 181. If the person does not have the energy or is not able to think clearly enough to investigate available sources of help, the first aider should offer to assist with this.

- Essential
- Important
- Don't know/Depends
- Unimportant
- Should not be included

* 182. If the person finds it overwhelming to decide who to see for their symptoms, the first aider should offer to assist with this.

- Essential
- Important
- Don't know/Depends
- Unimportant
- Should not be included

* 183. The first aider should know about the various types of treatment that have evidence of effectiveness for depression.

- Essential
- Important
- Don't know/Depends
- Unimportant
- Should not be included

* 184. The first aider should know about what services are available in their local area.

- Essential
- Important
- Don't know/Depends
- Unimportant
- Should not be included

* 185. The first aider should tell the person about the way treatment might help.

- Essential
- Important
- Don't know/Depends
- Unimportant
- Should not be included

186. Please provide any additional items or comments related to this section.

Help-seeking (cont)

Please rate how important (from ‘essential’ to ‘should not be included’) you think it is that each statement be included in the guidelines.

Please keep our definitions in mind when responding.

The first appointment with the health professional

* 187. The first aider should offer to make the first appointment for the person.

- Essential
- Important
- Don't know/Depends
- Unimportant
- Should not be included

* 188. If person is reluctant to get professional help, the first aider should offer to make the first appointment for the person.

- Essential
- Important
- Don't know/Depends
- Unimportant
- Should not be included

* 189. The first aider should offer to accompany the person to the appointment with the health professional.

- Essential
- Important
- Don't know/Depends
- Unimportant
- Should not be included

* 190. If person is reluctant to get professional help, the first aider should offer to accompany them to the appointment.

- Essential
- Important
- Don't know/Depends
- Unimportant
- Should not be included

* 191. If the first aider accompanies the person to the appointment, they must not take over completely, because a person with depression needs to make their own decisions as much as possible.

- Essential
- Important
- Don't know/Depends
- Unimportant
- Should not be included

192. Please provide any additional items or comments related to this section.

Updating the Mental Health First Aid Guidelines for Helping someone with Depression

Help-seeking (cont)

Please rate how important (from ‘essential’ to ‘should not be included’) you think it is that each statement be included in the guidelines.

Please keep our definitions in mind when responding.

Preparing for the appointment with the health professional

* 193. The first aider should avoid labelling the person’s behaviours or feelings as symptoms of depression when talking to them about seeking help.

- Essential
- Important
- Don't know/Depends
- Unimportant
- Should not be included

* 194. The first aider should ***encourage the person to make a list*** of symptoms to discuss with the health professional at their first appointment.

- Essential
- Important
- Don't know/Depends
- Unimportant
- Should not be included

* 195. The first aider should ***offer to help the person make a list*** of symptoms to discuss with the health professional at their first appointment.

- Essential
- Important
- Don't know/Depends
- Unimportant
- Should not be included

* 196. If the person agrees, the first aider should offer to call the person’s health professional in advance to tell them about the person's symptoms.

- Essential
- Important
- Don't know/Depends
- Unimportant
- Should not be included

* 197. If the first aider accompanies the person to the appointment, they should take a list of the person’s symptoms with them for discussion with the health professional.

- Essential
- Important
- Don't know/Depends
- Unimportant
- Should not be included

* 198. The first aider should ***encourage the person to make a list*** of questions they have to discuss with the health professional at their first appointment.

- Essential
- Important
- Don't know/Depends
- Unimportant
- Should not be included

* 199. The first aider should ***offer to help the person make a list*** of questions they have to discuss with the health professional at their first appointment.

- Essential
- Important
- Don't know/Depends
- Unimportant
- Should not be included

200. Please provide any additional items or comments related to this section.

Updating the Mental Health First Aid Guidelines for Helping someone with Depression

Help-seeking (cont)

Please rate how important (from ‘essential’ to ‘should not be included’) you think it is that each statement be included in the guidelines.

Please keep our definitions in mind when responding.

After the appointment

* 201. If the first aider does not accompany the person to their appointment, they should follow-up with the person to make sure they got appropriate professional help.

- Essential
- Important
- Don't know/Depends
- Unimportant
- Should not be included

* 202. If the first aider does not accompany the person to their appointment, they should ask how the appointment went.

- Essential
- Important
- Don't know/Depends
- Unimportant
- Should not be included

* 203. The first aider should know that depression is often not recognised by health professionals and that it may take some time to get a diagnosis. If this is the case, the first aider should encourage the person not to give up.

- Essential
- Important
- Don't know/Depends
- Unimportant
- Should not be included

* 204. The first aider should know that it may take some time to find a health professional with whom the person is able to establish a good relationship. If this is the case, the first aider should encourage the person not to give up.

- Essential
- Important
- Don't know/Depends
- Unimportant
- Should not be included

* 205. If the first aider finds a resource they think might help the person, they should leave it somewhere the person will find it rather than give it to them directly.

- Essential
- Important
- Don't know/Depends
- Unimportant
- Should not be included

* 206. The first aider should tell the person about options for getting evidence-based online or telephone mental health services.

- Essential
- Important
- Don't know/Depends
- Unimportant
- Should not be included

* 207. The first aider should be aware that the person may have special requirements with regards to seeking help for mental health problems due to their cultural background or religious beliefs.

- Essential
- Important
- Don't know/Depends
- Unimportant
- Should not be included

208. Please provide any additional items or comments related to this section.

Updating the Mental Health First Aid Guidelines for Helping someone with Depression

Self-help strategies

Please rate how important (from 'essential' to 'should not be included') you think it is that each statement be included in the guidelines.

Please keep our definitions in mind when responding.

* 209. The first aider should know what self-help strategies have evidence to support their effectiveness and should encourage the person to use these strategies.

- Essential
- Important
- Don't know/Depends
- Unimportant
- Should not be included

* 210. If the person shows an interest in using self-help strategies, the first aider should encourage the person to use evidence-based strategies.

- Essential
- Important
- Don't know/Depends
- Unimportant
- Should not be included

* 211. The first aider needs to know that the person's ability and desire to use self-help strategies will depend on their interest and the severity of their depression.

- Essential
- Important
- Don't know/Depends
- Unimportant
- Should not be included

* 212. The first aider should not be too forceful when trying to encourage the person to use evidence-based self-help strategies.

- Essential
- Important
- Don't know/Depends
- Unimportant
- Should not be included

* 213. The first aider can support the person by offering to do some evidence-based self-help activities with them, e.g. exercise.

- Essential
- Important
- Don't know/Depends
- Unimportant
- Should not be included

* 214. If the person is interested in self-help strategies, the first aider should provide them with a range of information about evidence-based self-help strategies.

- Essential
- Important
- Don't know/Depends
- Unimportant
- Should not be included

* 215. The first aider should tell the person that online screening tests and checklists are available to help them decide if they are depressed.

- Essential
- Important
- Don't know/Depends
- Unimportant
- Should not be included

* 216. The first aider should discourage the person from using alcohol or other drugs to feel better.

- Essential
- Important
- Don't know/Depends
- Unimportant
- Should not be included

217. Please provide any additional items or comments related to this section.

Updating the Mental Health First Aid Guidelines for Helping someone with Depression

What to do if the person doesn't want help

Please rate how important (from ‘essential’ to ‘should not be included’) you think it is that each statement be included in the guidelines.

Please keep our definitions in mind when responding.

* 218. The first aider should know the barriers to seeking treatment for depression, e.g. the person feels like no one can really help.

- Essential
- Important
- Don't know/Depends
- Unimportant
- Should not be included

* 219. If the person refuses to seek or accept professional help, the first aider should:

	Essential	Important	Depends/Don't know	Unimportant	Should not be included
Attempt to enlist the help of the person’s family, friends or workmates.					
Attempt to enlist the help of a health professional.					

	Essential	Important	Depends/Don't know	Unimportant	Should not be included
Seek advice from a health professional or help line about what to do.	○	○	○	○	○
Seek advice from someone who has experienced depression and received treatment.	○	○	○	○	○
Encourage the person to find out more about depression.	○	○	○	○	○
Encourage the person to attend a support group.	○	○	○	○	○
Encourage the person to use self-help strategies.	○	○	○	○	○
Let the person know that they will keep checking in on them because they are concerned about the person.	○	○	○	○	○
Suggest to the person that they try evidence-based alternative treatments, e.g. exercise.	○	○	○	○	○
Use deception, coercion, threats, or whatever is necessary to ensure professional help is received.	○	○	○	○	○
Not use deception, coercion or threats to ensure professional help is received.	○	○	○	○	○
Not suggest that they get professional help again unless the person asks about it.	○	○	○	○	○

* 220. If the person refuses to seek or accept face-to-face professional help, the first aider should encourage them to get online professional help.

- Essential
- Important
- Don't know/Depends
- Unimportant
- Should not be included

221. Please provide any additional items or comments related to this section.

Updating the Mental Health First Aid Guidelines for Helping someone with Depression

What to do if the person doesn't want help (cont)

Please rate how important (from ‘essential’ to ‘should not be included’) you think it is that each statement be included in the guidelines.

Please keep our definitions in mind when responding.

* 222. If the person lives in a family, the first aider should label the person's depression as a 'family problem' and suggest that the whole family attends therapy. This will take the focus off the depressed person.

- Essential
- Important
- Don't know/Depends
- Unimportant
- Should not be included

* 223. The first aider should contact the person’s doctor directly to explain the situation. Then they should encourage the person to seek help for the physical symptoms of depression so the doctor can raise the issue of depression in that context.

- Essential
- Important
- Don't know/Depends
- Unimportant
- Should not be included

* 224. The first aider should not go behind the person’s back to seek professional help.

- Essential
- Important
- Don't know/Depends
- Unimportant
- Should not be included

* 225. The first aider should find out if there are specific reasons why the person does not want to seek professional help (e.g. concerns about finances, not having a doctor they like, or being worried they will be sent to hospital), as sometimes such reasons are based on mistaken beliefs, or can be overcome with help.

- Essential
- Important
- Don't know/Depends
- Unimportant
- Should not be included

* 226. If, in spite of the first aider’s efforts, the person is still unwilling to seek or accept professional help, the first aider must respect their wishes.

- Essential
- Important
- Don't know/Depends
- Unimportant
- Should not be included

* 227. If the first aider does want to seek professional advice regarding options, they should always inform the person of their intended actions.

- Essential
- Important
- Don't know/Depends
- Unimportant
- Should not be included

* 228. Sometimes the person may need time to accept the need for treatment. The first aider should gently and respectfully persist in trying to get the person to seek help.

- Essential
- Important
- Don't know/Depends
- Unimportant
- Should not be included

* 229. The first aider should let the person know that if they change their mind in the future about seeking help, they can contact the first aider.

- Essential
- Important
- Don't know/Depends
- Unimportant
- Should not be included

* 230. The first aider should know that if they try to force or pressure the person into seeking help it could turn the person off seeking help altogether.

- Essential
- Important
- Don't know/Depends
- Unimportant
- Should not be included

231. Please provide any additional items or comments related to this section.

Updating the Mental Health First Aid Guidelines for Helping someone with Depression

What to do if the person doesn't want help (cont)

Please rate how important (from ‘essential’ to ‘should not be included’) you think it is that each statement be included in the guidelines.

Please keep our definitions in mind when responding.

* 232. If the person is reluctant to admit there is a problem, the first aider should be gently persistent with their concerns.

- Essential
- Important
- Don't know/Depends
- Unimportant
- Should not be included

* 233. If the person acknowledges that they have a problem, but does not want to get help, the first aider should be gently insistent that the person take steps to address their depression.

- Essential
- Important
- Don't know/Depends
- Unimportant
- Should not be included

* 234. The first aider should not push the person into seeking professional help before they are ready, unless there is a specific risk of harm to self or others.

- Essential
- Important
- Don't know/Depends
- Unimportant
- Should not be included

* 235. If the person is unwilling or unable to recognise that there’s a serious problem, the first aider should consider staging an intervention with the person and their friends and family. (***An intervention*** is when a group of people who care about the person meet with the person to point out the behaviours that are causing problems and ask them to get help. An intervention includes a discussion around the consequences for the person if they do not get help for their depression.)

- Essential
- Important
- Don't know/Depends
- Unimportant
- Should not be included

* 236. If the person is unwilling or unable to recognise that there’s a serious problem, the first aider should ***not*** stage an intervention with the person and their friends and family.

- Essential
- Important
- Don't know/Depends
- Unimportant
- Should not be included

237. Please provide any additional items or comments related to this section.

Updating the Mental Health First Aid Guidelines for Helping someone with Depression

Difficulties the first aider may encounter

Please rate how important (from ‘essential’ to ‘should not be included’) you think it is that each statement be included in the guidelines.

Please keep our definitions in mind when responding.

* 238. If the person becomes angry during the conversation, the first aider should:

	Essential	Important	Depends/Don't know	Unimportant	Should not be included
Stay calm	◯	◯	◯	◯	◯
Acknowledge the anger.	◯	◯	◯	◯	◯
Not make assumptions about the cause of their anger.	◯	◯	◯	◯	◯

* 239. If the person appears irrational, the first aider should not try to talk the person out of their thoughts or feelings.

◯ Essential

◯ Important

◯ Don't know/Depends

◯ Unimportant

◯ Should not be included

* 240. The first aider should not agree with distorted negative thoughts, as these are a symptom of depression.

◯ Essential

◯ Important

◯ Don't know/Depends

◯ Unimportant

◯ Should not be included

* 241. To help the person, the first aider should point out distorted negative thinking without being critical or disapproving.

◯ Essential

◯ Important

◯ Don't know/Depends

◯ Unimportant

◯ Should not be included

* 242. If the person is unrelentingly pessimistic, the first aider should try to point out the positive things that are happening.

- Essential
- Important
- Don't know/Depends
- Unimportant
- Should not be included

* 243. If the first aider is feeling upset or worn out after helping the person they should:

	Essential	Important	Depends/Don't know	Unimportant	Should not be included
Find someone to talk to about their feelings without sharing the personal details of the person they were helping.	○	○	○	○	○
Use evidence-based self-care strategies.	○	○	○	○	○

244. Please provide any additional items or comments related to this section.

Updating the Mental Health First Aid Guidelines for Helping someone with Depression

What to do if there are concerns for safety

Please rate how important (from ‘essential’ to ‘should not be included’) you think it is that each statement be included in the guidelines.

Please keep our definitions in mind when responding.

Non-verbal communication

* 245. The first aider should respect the person’s right not to seek help unless the first aider believes that the person is at risk of harming themselves or others.

- Essential
- Important
- Don't know/Depends
- Unimportant
- Should not be included

* 246. If the person is at risk of harming themselves or others, the first aider should:

	Essential	Important	Depends/Don't know	Unimportant	Should not be included
Ask the person if it’s okay to talk to someone else about their concerns for the person's safety.	○	○	○	○	○
Tell the person that the first aider needs to get someone else involved even without their permission.	○	○	○	○	○
Seek help on their behalf even without the person's permission.	○	○	○	○	○

* 247. The first aider should be aware of the Mental Health First Aid Guidelines for how to assist someone with suicidal thoughts or behaviours.

- Essential
- Important
- Don't know/Depends
- Unimportant
- Should not be included

* 248. The first aider should seek immediate professional help if the person is experiencing hallucinations or delusions.

- Essential
- Important
- Don't know/Depends
- Unimportant
- Should not be included

249. Please provide any additional items or comments related to this section.

Updating the Mental Health First Aid Guidelines for Helping someone with Depression

Thank you!

Thank you for sharing your expertise and time with us.

If anything in this survey has caused you distress and you would like to talk with someone about it you can contact the appropriate crisis help line below:

Australia: Lifeline on 13 11 14

Canada: National Suicide prevention Lifeline on 1800 273 TALK (8255)

Denmark: Suicide hotline 70 201 201

Finland: SOS Crisis Centre 010 195 202

The Netherlands: Suicide hotline 113Online

New Zealand: Lifeline Aotearoa on 0800 543 354

Republic of Ireland: Samaritans on 116 123

Sweden: Suicide hotline 020 22 00 60

UK: Samaritans on 116 123

USA: National Suicide prevention Lifeline on 1800 273 TALK (8255)

Round 2: Updating the Mental Health First Aid Guidelines for Helping someone with Depression

Information about this research

Purpose of this research

The aim of this current research project is to update the mental health first aid guidelines for how a member of the public should give assistance to a person who is experiencing depression. These guidelines are being developed for high income Western countries.

How this questionnaire was developed

The statements in this questionnaire were derived from the results of the Round 1 survey. You will note that each statement is marked as either a NEW or RERATE item. New items were derived from the comments provided in the first survey. An item is rerated when:

- 70%–79% of panel members from both expert groups rated it as essential or important, OR
- 70%-79% of one group rated it as essential or important and 80%-100% of the other group did.

Consent to participate

It is important for you to know that participation in this study is completely voluntary. You are not under any obligation to participate and you can withdraw at any time.

We would like to thank you for your time and effort, and encourage you to provide us with feedback on this process.

Definitions used in this survey

Mental health first aid is the help offered to a person developing a mental health problem, experiencing a worsening of an existing mental health problem, or in a mental health crisis. The first aid is given until appropriate professional help is received or until the crisis resolves.

The person refers to the person who the mental health first aider is concerned may be experiencing depression.

The first aider refers to a concerned family member, friend, work colleague or member of the community, who provides help to a person who may be experiencing depression.

Mental health professional refers to a trained health professional who is treating/working with the person. This could be a psychologist, mental health nurse or psychiatrist. It could also be another health professional who has specialist mental health training, e.g. social worker, occupational therapist or GP.

Instructions

Please complete the questionnaire by rating each statement ***according to how important you believe it is for inclusion in the guidelines*** for providing mental health first aid to a person who may be experiencing depression. Please keep in mind that the guidelines will be used by the general public. The statements need to be rated according to their importance for someone ***without a counselling or clinical background***.

This questionnaire should take approximately 45 minutes to complete. You can complete the survey in two or more sittings. **Your answers are saved when you click 'Next' at the bottom of a page.** This marks your page and you can begin again at a later date on the next page. ***Please be aware that once you have logged on and started responding you must complete the questionnaire on the same computer.***

Round 2: Updating the Mental Health First Aid Guidelines for Helping someone with Depression

Information about you

* 1. What is your name? (This allows us to determine who has completed the Round 2 survey and is therefore eligible to participate in Round 3. Your name will be deleted from your data when the project is complete).

* 2. Are you a Mental Health First Aid **Instructor**, i.e. do you train others in Mental Health First Aid.

- Yes
- No

Overview of the study

Section 1: Learning about depression
Section 2: Approaching someone who may be experiencing depression
Section 3: Being supportive
Section 4: Communicating effectively
Section 5: Encouraging help seeking
Section 6: Encouraging self-help strategies
Section 7: What to do if they do not want help
Section 8: Concerns for safety

Round 2: Updating the Mental Health First Aid Guidelines for Helping someone with Depression

Learning about depression

Please rate how important (from 'essential' to 'should not be included') you think it is that each statement be included in the guidelines.

Please keep our definitions in mind when responding.

* 3. The first aider should learn more about depression by:

	Essential	Important	Depends/Don't know	Unimportant	Should not be included
a. Seeking advice from people who have experienced depression (Rerate)	◯	◯	◯	◯	◯
b. Seeking advice from people who have experienced **and recovered from** depression (New)	◯	◯	◯	◯	◯
c. Seeking advice from a mental health professional (Rerate)	◯	◯	◯	◯	◯

* 4. If assisting someone from a cultural background that is different from the first aider's, the first aider should learn about how depression symptoms may manifest in people from the person's cultural background. (New)

◯ Essential

◯ Important

◯ Don't know/Depends

◯ Unimportant

◯ Should not be included

Round 2: Updating the Mental Health First Aid Guidelines for Helping someone with Depression

Approaching someone who may be experiencing depression

Please rate how important (from ‘essential’ to ‘should not be included’) you think it is that each statement be included in the guidelines.

Please keep our definitions in mind when responding.

If the first aider notices signs or symptoms of depression

* 5. The first aider should consider whether they are the best person to approach the person or whether somebody else might be more appropriate. (New)

- Essential
- Important
- Don't know/Depends
- Unimportant
- Should not be included

* 6. The first aider should not assume that the person's symptoms are due to depression. (New)

- Essential
- Important
- Don't know/Depends
- Unimportant
- Should not be included

* 7. If the first aider notices changes in the mood, behaviour or daily functioning of someone they know, they should approach the person about their concerns. (New)

- Essential
- Important
- Don't know/Depends
- Unimportant
- Should not be included

* 8. If the first aider thinks someone may be depressed, they should try to spend time with the person and gently bring up their concerns with them, e.g. mention that the person seems down today. (New)

- Essential
- Important
- Don't know/Depends
- Unimportant
- Should not be included

* 9. The first aider should be open to any opportunity that presents itself to talk about their concerns with the person. (New)

- Essential
- Important
- Don't know/Depends
- Unimportant
- Should not be included

* 10. If the first aider cannot find the ideal place and time to talk, they should not let this delay talking to the person. (New)

- Essential
- Important
- Don't know/Depends
- Unimportant
- Should not be included

Preparing for the conversation

* 11. The first aider should plan in advance what they are going to say to the person. (New)

- Essential
- Important
- Don't know/Depends
- Unimportant
- Should not be included

Starting a conversation

* 12. The first aider should ask the person if they would like to talk to the first aider about how they are feeling. (New)

- Essential
- Important
- Don't know/Depends
- Unimportant
- Should not be included

* 13. The first aider should ask the person if they are willing to talk to the first aider, or if they would rather speak to someone else. (New)

- Essential
- Important
- Don't know/Depends
- Unimportant
- Should not be included

Round 2: Updating the Mental Health First Aid Guidelines for Helping someone with Depression

Approaching someone who may be experiencing depression (cont)

Please rate how important (from ‘essential’ to ‘should not be included’) you think it is that each statement be included in the guidelines.

Please keep our definitions in mind when responding.

Giving the person information

* 14. The first aider should tell the person that depression is common. (Rerate)

- Essential
- Important
- Don't know/Depends
- Unimportant
- Should not be included

* 15. The first aider should let the person know that there are online screening tools for depression. (New)

- Essential
- Important
- Don't know/Depends
- Unimportant
- Should not be included

* 16. Before offering information to the person, the first aider should consider the person's literacy and ability to understand the information. (New)

- Essential
- Important
- Don't know/Depends
- Unimportant
- Should not be included

* 17. The first aider should not overwhelm the person with too much information or too many resources. (New)

- Essential
- Important
- Don't know/Depends
- Unimportant
- Should not be included

When talking to the person

* 18. The first aider should ask the person if anyone else knows how they are feeling. (New)

- Essential
- Important
- Don't know/Depends
- Unimportant
- Should not be included

* 19. The first aider should ask the person if they have spoken to anyone else about how they are feeling. (New)

- Essential
- Important
- Don't know/Depends
- Unimportant
- Should not be included

* 20. The first aider should focus on how the person is feeling rather than on the possibility that the person may have depression. (New)

- Essential
- Important
- Don't know/Depends
- Unimportant
- Should not be included

* 21. The first aider should focus on the changes they have noticed in the person rather than on the possibility that the person may have depression. (New)

- Essential
- Important
- Don't know/Depends
- Unimportant
- Should not be included

* 22. The first aider should not attempt to diagnose the person. (New)

- Essential
- Important
- Don't know/Depends
- Unimportant
- Should not be included

* 23. The first aider should not use language related to a potential diagnosis when talking with the person. (New)

- Essential
- Important
- Don't know/Depends
- Unimportant
- Should not be included

* 24. The first aider should be aware that the person may deny that they are experiencing changes in mood, behaviour or daily functioning. (New)

- Essential
- Important
- Don't know/Depends
- Unimportant
- Should not be included

* 25. The first aider should let the person know in advance that they will intervene and seek professional help for the person if they ever believe the person's life may be in danger. (Rerate)

- Essential
- Important
- Don't know/Depends
- Unimportant
- Should not be included

Round 2: Updating the Mental Health First Aid Guidelines for Helping someone with Depression

Being supportive

Please rate how important (from 'essential' to 'should not be included') you think it is that each statement be included in the guidelines.

Please keep our definitions in mind when responding.

Understanding depression

* 26. The first aider should know that those who haven't experienced depression cannot fully understand what it is like for those who have. (Rerate)

- Essential
- Important
- Don't know/Depends
- Unimportant
- Should not be included

* 27. The first aider should tell the person that although their experience is very personal and painful, they are not alone. (New)

- ◯ Essential
- ◯ Important
- ◯ Don't know/Depends
- ◯ Unimportant
- ◯ Should not be included

* 28. The first aider should be aware that the person's thoughts, feelings and beliefs represent their own reality and the first aider should be prepared to accept these without question. (New)

- ◯ Essential
- ◯ Important
- ◯ Don't know/Depends
- ◯ Unimportant
- ◯ Should not be included

Offering support

* 29. The first aider should know that allowing the person to talk about how they are feeling can help them feel better, not worse. (New)

- ◯ Essential
- ◯ Important
- ◯ Don't know/Depends
- ◯ Unimportant
- ◯ Should not be included

* 30. The first aider should know that the person may become obsessive in talking about their feelings, making them seem self-absorbed. (Rerate)

- ◯ Essential
- ◯ Important
- ◯ Don't know/Depends
- ◯ Unimportant
- ◯ Should not be included

* 31. The first aider should not use a 'tough-love' approach to try and make the person better, e.g. the first aider telling the person they will not spend time with them until they get better or get professional help. (Rerate)

- ◯ Essential
- ◯ Important
- ◯ Don't know/Depends
- ◯ Unimportant
- ◯ Should not be included

* 32. If the first aider does not feel that they are able to help the person, they should ask someone else to take on the first aider role. (New)

- ◯ Essential
- ◯ Important
- ◯ Don't know/Depends
- ◯ Unimportant
- ◯ Should not be included

* 33. The first aider should let the person know that they will not abandon them. (Rerate)

- ◯ Essential
- ◯ Important
- ◯ Don't know/Depends
- ◯ Unimportant
- ◯ Should not be included

* 34. The first aider should be upfront with the person about the limitations of their role as a first aider, e.g. they are not a counsellor. (New)

- ◯ Essential
- ◯ Important
- ◯ Don't know/Depends
- ◯ Unimportant
- ◯ Should not be included

* 35. The first aider should be honest with the person about what help they can and are willing to offer. (New)

- Essential
- Important
- Don't know/Depends
- Unimportant
- Should not be included

* 36. If the first aider is particularly concerned about the person, they should**ask the person whether they would like the first aider to help organise** close friends or family members to make regular contact with the person, e.g. to provide practical help, have a coffee, simply to say hello. (New)

- Essential
- Important
- Don't know/Depends
- Unimportant
- Should not be included

* 37. If the first aider is particularly concerned about the person, they should**suggest that the person contacts friends or family members** and asks them to make regular contact, e.g. to provide practical help, have a coffee, simply to say hello. (New)

- Essential
- Important
- Don't know/Depends
- Unimportant
- Should not be included

* 38. If the person lives alone and finds this difficult, the first aider should discuss options with the person so that they are not alone, e.g. having someone stay with them, arranging for friends to take turns staying with them, or inviting them to stay with the first aider. (New)

- Essential
- Important
- Don't know/Depends
- Unimportant
- Should not be included

Being supportive (cont)

Please rate how important (from ‘essential’ to ‘should not be included’) you think it is that each statement be included in the guidelines.

Please keep our definitions in mind when responding.

Messages about recovery

* 39. The first aider should know that recovery, for the most part, must be led by the person. (New)

- Essential
- Important
- Don't know/Depends
- Unimportant
- Should not be included

* 40. The first aider should offer **emotional support and hope** of a more positive future in whatever form the depressed person will accept. (Rerate)

- Essential
- Important
- Don't know/Depends
- Unimportant
- Should not be included

* 41. The first aider should offer **emotional support** of a more positive future in whatever form the depressed person will accept. (New)

- Essential
- Important
- Don't know/Depends
- Unimportant
- Should not be included

* 42. The first aider should offer **hope** of a more positive future in whatever form the depressed person will accept. (New)

- Essential
- Important
- Don't know/Depends
- Unimportant
- Should not be included

* 43. The first aider should let the person know that getting better takes time, but that it will happen. (New)

- Essential
- Important
- Don't know/Depends
- Unimportant
- Should not be included

* 44. The first aider should let the person know that things won't feel this bad forever. (New)

- Essential
- Important
- Don't know/Depends
- Unimportant
- Should not be included

* 45. The first aider should tell the person that they may be able to resolve their problems when they are feeling better. (New)

- Essential
- Important
- Don't know/Depends
- Unimportant
- Should not be included

* 46. The first aider should tell the person that they believe the person can resolve their problems with the support of others. (New)

- Essential
- Important
- Don't know/Depends
- Unimportant
- Should not be included

* 47. The first aider should tell the person that they believe the person can resolve their problems with professional support. (New)

- Essential
- Important
- Don't know/Depends
- Unimportant
- Should not be included

* 48. The first aider should let the person know that they are not weak or a failure because they have depression, and that they don't think less of them as a person. (Rerate)

- Essential
- Important
- Don't know/Depends
- Unimportant
- Should not be included

49. If the person says that they feel they are a weak person or a failure, the first aider should let the person know that:

	Essential	Important	Don't know/Depends	Unimportant	Should not be included
Strong and capable people can become depressed (New)	○	○	○	○	○
They don't think the person is weak or a failure (New)	○	○	○	○	○
They don't think less of them as a person (New)	○	○	○	○	○

Being supportive (cont)

Please rate how important (from ‘essential’ to ‘should not be included’) you think it is that each statement be included in the guidelines.

Please keep our definitions in mind when responding.

Offering practical assistance

* 50. The first aider should provide similar support as they would if the person had a physical illness, e.g. sending a 'get-well' card or flowers. (New)

- Essential
- Important
- Don't know/Depends
- Unimportant
- Should not be included

* 51. The first aider should let the person know that stress is a risk factor for depression and encourage them to find ways to reduce stress in their life. (Rerate)

- Essential
- Important
- Don't know/Depends
- Unimportant
- Should not be included

* 52. The first aider should ask the person if stress is a problem for them and, if it is, encourage them to find ways to reduce stress in their life. (New)

- Essential
- Important
- Don't know/Depends
- Unimportant
- Should not be included

* 53. The first aider should ask the person whether something has happened to them recently that is contributing to how they are feeling. (New)

- Essential
- Important
- Don't know/Depends
- Unimportant
- Should not be included

* 54. The first aider should explore with the person how their symptoms affect their daily life. (New)

- Essential
- Important
- Don't know/Depends
- Unimportant
- Should not be included

* 55. The first aider should ask the person how they are feeling on a scale of 1 to 10. (New)

- Essential
- Important
- Don't know/Depends
- Unimportant
- Should not be included

* 56. The first aider should ask the person what practical assistance they need. (Rerate)

- Essential
- Important
- Don't know/Depends
- Unimportant
- Should not be included

* 57. The first aider should ask the person if they would like any practical assistance with tasks, but should be careful not to take over or encourage dependency. (Rerate)

- Essential
- Important
- Don't know/Depends
- Unimportant
- Should not be included

* 58. If the person is finding it difficult getting practical tasks done, the first aider should work together with the person on this. (New)

- Essential
- Important
- Don't know/Depends
- Unimportant
- Should not be included

* 59. The first aider should encourage the person to participate in chores or activities by giving the person incentives to participate. (New)

- Essential
- Important
- Don't know/Depends
- Unimportant
- Should not be included

* 60. If the person is finding it difficult to make decisions, the first should work together with the person on this. (New)

- Essential
- Important
- Don't know/Depends
- Unimportant
- Should not be included

* 61. If the person is having trouble making decisions, the first aider should ask the person whether there are any decisions they would like the first aider to make for them. (New)

- Essential
- Important
- Don't know/Depends
- Unimportant
- Should not be included

Round 2: Updating the Mental Health First Aid Guidelines for Helping someone with Depression

Being supportive (cont)

Please rate how important (from ‘essential’ to ‘should not be included’) you think it is that each statement be included in the guidelines.

Please keep our definitions in mind when responding.

What not to do or say

* 62. The first aider should avoid using the words "I know how you feel" or “I understand”. (Rerate)

- Essential
- Important
- Don't know/Depends
- Unimportant
- Should not be included

* 63. Unless the first aider has personal experience with depression, they should avoid using the words "I know how you feel" or “I understand”. (New)

- Essential
- Important
- Don't know/Depends
- Unimportant
- Should not be included

If the first aider is affected by the conversation

* 64. If the person’s behaviour is having a negative effect on the first aider, the first aider should recognise their own feelings and deal with these separately. (New)

- Essential
- Important
- Don't know/Depends
- Unimportant
- Should not be included

* 65. If the person’s behaviour is having a negative effect on the first aider, the first aider should discuss their feelings with a professional. (New)

- Essential
- Important
- Don't know/Depends
- Unimportant
- Should not be included

* 66. If the person’s behaviour is having a negative effect on others, the first aider should let the person know in order to raise their awareness. (New)

- Essential
- Important
- Don't know/Depends
- Unimportant
- Should not be included

* 67. If the first aider is frustrated because of their own limitations as a helper, they should let the person know this. (New)

- Essential
- Important
- Don't know/Depends
- Unimportant
- Should not be included

* 68. If the first aider feels frustrated, they should let the person know they are frustrated with the challenges that the person is experiencing, not with them. (New)

- Essential
- Important
- Don't know/Depends
- Unimportant
- Should not be included

* 69. The first aider should not accept abuse or compromise their own mental health when helping the person. (New)

- Essential
- Important
- Don't know/Depends
- Unimportant
- Should not be included

Round 2: Updating the Mental Health First Aid Guidelines for Helping someone with Depression

Communicating effectively

Please rate how important (from ‘essential’ to ‘should not be included’) you think it is that each statement be included in the guidelines.

Please keep our definitions in mind when responding.

Talking to the person

* 70. The first aider should not use the term 'depression' unless the person uses the term themselves. (New)

- Essential
- Important
- Don't know/Depends
- Unimportant
- Should not be included

* 71. The first aider should use the same terminology that the person uses when discussing their experience, e.g. if they say they are "moody" or "down", use these terms rather than "depressed", or vice versa. (Rerate)

- Essential
- Important
- Don't know/Depends
- Unimportant
- Should not be included

* 72. The first aider should use the same terminology that the person uses when discussing their experience, **except if the person uses unhelpful or stigmatising language** (New)

- Essential
- Important
- Don't know/Depends
- Unimportant
- Should not be included

* 73. Where possible, the first aider should allow the person to guide the duration of the conversation. (New)

- Essential
- Important
- Don't know/Depends
- Unimportant
- Should not be included

Listening to the person

* 74. The first aider should encourage the person to talk about their thoughts, feelings, symptoms and any other problems they are experiencing. (Rerate)

- Essential
- Important
- Don't know/Depends
- Unimportant
- Should not be included

* 75. The first aider should encourage the person to talk about their thoughts, feelings, symptoms and any other problems they are experiencing, **when the person is ready**. (New)

- Essential
- Important
- Don't know/Depends
- Unimportant
- Should not be included

<u>If the person find it difficult to talk</u>

* 76. If the person finds it difficult to discuss their thoughts and feelings openly, the first aider should suggest an activity that may make it easier for them to talk, e.g. have a cup of tea, go for a walk. (New)

- Essential
- Important
- Don't know/Depends
- Unimportant
- Should not be included

* 77. If the person finds it difficult to discuss their thoughts and feelings openly with the first aider, the first aider should let the person know about available services where they can talk to someone else, e.g. telephone counselling service. (New)

- Essential
- Important
- Don't know/Depends
- Unimportant
- Should not be included

* 78. If the person does not want to talk to the first aider, the first aider should suggest they talk to someone else. (New)

- Essential
- Important
- Don't know/Depends
- Unimportant
- Should not be included

Communicating effectively (cont)

Please rate how important (from ‘essential’ to ‘should not be included’) you think it is that each statement be included in the guidelines.

Please keep our definitions in mind when responding.

Non-verbal communication

* 79. The first aider should use the following non-verbal skills to reinforce their non-judgmental communication:

	Essential	Important	Depends/Don't know	Unimportant	Should not be included
a. If it is safe, sit down, even if the person is standing. This may seem less threatening. (Rerate)	○	○	○	○	○
b. Sit alongside the person and angled towards them, rather than directly opposite them. (Rerate)	○	○	○	○	○

Cultural considerations when communicating with the person

* 80. If cultural differences are interfering with the first aider's ability to help the person, they should:

	Essential	Important	Depends/Don't know	Unimportant	Should not be included
a. Seek advice from someone from the person's cultural background. (Rerate)	○	○	○	○	○
b. Seek advice from someone from the person's cultural background, **with the person's permission.** (New)	○	○	○	○	○
c. Talk to a mental health service that specialises in working with people from different cultural backgrounds. (New)	○	○	○	○	○

Round 2: Updating the Mental Health First Aid Guidelines for Helping someone with Depression

Help-seeking

Please rate how important (from ‘essential’ to ‘should not be included’) you think it is that each statement be included in the guidelines.

Please keep our definitions in mind when responding.

When to encourage help-seeking

* 81. The first aider should encourage the person to get professional help. (Rerate)

- ○ Essential
- ○ Important
- ○ Don't know/Depends
- ○ Unimportant
- ○ Should not be included

* 82. The first aider should encourage the person to get professional help***as early as possible***. (Rerate)

- Essential
- Important
- Don't know/Depends
- Unimportant
- Should not be included

* 83. If the person has been in a low mood for two weeks or more, the first aider should get professional help. (New)

- Essential
- Important
- Don't know/Depends
- Unimportant
- Should not be included

Knowledge about professional help

* 84. The first aider should know about the local pathways to professional help, e.g. referral from a GP in order to see a specialist. (New)

- Essential
- Important
- Don't know/Depends
- Unimportant
- Should not be included

* 85. The first aider should have some general knowledge about the types of treatment that can be helpful for depression. (New)

- Essential
- Important
- Don't know/Depends
- Unimportant
- Should not be included

Help-seeking (cont)

Please rate how important (from 'essential' to 'should not be included') you think it is that each statement be included in the guidelines.

Please keep our definitions in mind when responding.

How to assist the person with help-seeking

* 86. The first aider should talk about professional help seeking in a way that normalises it, e.g. talk about it as a natural action to take, explain that mental health problems are common and treatable. (New)

- ◯ Essential
- ◯ Important
- ◯ Don't know/Depends
- ◯ Unimportant
- ◯ Should not be included

* 87. The first aider should discuss with the person whether they need professional help or not. (New)

- ◯ Essential
- ◯ Important
- ◯ Don't know/Depends
- ◯ Unimportant
- ◯ Should not be included

* 88. The first aider should ask the person whether they think they would benefit from professional help. (New)

- ◯ Essential
- ◯ Important
- ◯ Don't know/Depends
- ◯ Unimportant
- ◯ Should not be included

* 89. If the person says they think they would benefit from professional help, the first aider should offer to help them organise this. (New)

- Essential
- Important
- Don't know/Depends
- Unimportant
- Should not be included

* 90. The first aider should offer to assist the person to investigate available sources of help. (Rerate)

- Essential
- Important
- Don't know/Depends
- Unimportant
- Should not be included

* 91. If the person does not have the energy or is not able to think clearly enough to investigate available sources of help, the first aider should offer to assist with this. (Rerate)

- Essential
- Important
- Don't know/Depends
- Unimportant
- Should not be included

92. The first aider should talk with the person about:

	Essential	Important	Don't know/Depends	Unimportant	Should not be included
The benefits of seeking professional help (New)					
The types of treatment available (New)					
Locally available sources of help (New)					

* 93. If the person shows an interest in knowing more about treatment, the first aider should share what they know. (New)

- Essential
- Important
- Don't know/Depends
- Unimportant
- Should not be included

* 94. The first aider should offer to support the person when they call to make an appointment with a mental health professional. (New)

- Essential
- Important
- Don't know/Depends
- Unimportant
- Should not be included

* 95. The first aider should avoid labelling the person's behaviours or feelings as symptoms of depression when talking to them about seeking help. (Rerate)

- Essential
- Important
- Don't know/Depends
- Unimportant
- Should not be included

Round 2: Updating the Mental Health First Aid Guidelines for Helping someone with Depression

Help-seeking (cont)

Please rate how important (from 'essential' to 'should not be included') you think it is that each statement be included in the guidelines.

Please keep our definitions in mind when responding.

Preparing for the appointment with the health professional

* 96. The first aider should ***encourage the person to make a list*** of symptoms to discuss with the health professional at their first appointment. (Rerate)

- Essential
- Important
- Don't know/Depends
- Unimportant
- Should not be included

* 97. The first aider should ***encourage the person to make a list*** of questions they have to discuss with the health professional at their first appointment. (Rerate)

- Essential
- Important
- Don't know/Depends
- Unimportant
- Should not be included

* 98. The first aider should offer any available resources that will help the person prepare for an appointment with a mental health professional. (New)

- Essential
- Important
- Don't know/Depends
- Unimportant
- Should not be included

* 99. The first aider should ask the person how much involvement they want the first aider to have with planning for and attending their appointment. (New)

- Essential
- Important
- Don't know/Depends
- Unimportant
- Should not be included

Self-help strategies

Please rate how important (from ‘essential’ to ‘should not be included’) you think it is that each statement be included in the guidelines.

Please keep our definitions in mind when responding.

* 100. Before suggesting self-help strategies, the first aider should ask the person what they are currently doing or what they have done in the past that they found helpful. (New)

- ◯ Essential
- ◯ Important
- ◯ Don't know/Depends
- ◯ Unimportant
- ◯ Should not be included

* 101. The first aider should encourage the person to use self-help strategies that have helped the person in the past. (New)

- ◯ Essential
- ◯ Important
- ◯ Don't know/Depends
- ◯ Unimportant
- ◯ Should not be included

* 102. If the person is interested in self-help strategies, the first aider should:

	Essential	Important	Don't know/Depends	Unimportant	Should not be included
a. Discuss with them a range of self-help strategies that might be helpful (New)	○	○	○	○	○
b. Encourage the person to use evidence-based strategies (Rerate)	○	○	○	○	○
c. Encourage the person to use appropriate strategies (New)	○	○	○	○	○
d. Encourage them to consult reputable sources about what is most likely to be helpful, e.g. health department sponsored website (New)	○	○	○	○	○

* 103. If the person is interested in self-help activities and it is appropriate to their relationship, the first aider should offer to do self-help activities with the person. (New)

- ○ Essential
- ○ Important
- ○ Don't know/Depends
- ○ Unimportant
- ○ Should not be included

Round 2: Updating the Mental Health First Aid Guidelines for Helping someone with Depression

What to do if the person doesn't want help

Please rate how important (from ‘essential’ to ‘should not be included’) you think it is that each statement be included in the guidelines.

Please keep our definitions in mind when responding.

* 104. If the person refuses to seek or accept professional help, the first aider should:

	Essential	Important	Depends/Don't know	Unimportant	Should not be included
a. Seek advice from a health professional or help line about what to do. (Rerate)	○	○	○	○	○
b. Encourage the person to find out more about depression. (Rerate)	○	○	○	○	○
c. Encourage the person to find out more about their mental health problems (New)	○	○	○	○	○
d. Encourage them to get online professional help, where this is available. (New)	○	○	○	○	○
e. Ask the person if they have had a bad experience with seeking help for their mental health problems. (New)	○	○	○	○	○
f. Ask the person whether they would like the first aider to check in on them (New)	○	○	○	○	○
g. Let the person know that they will keep checking in on them because they are concerned about the person. (Rerate)	○	○	○	○	○
h. Keep checking in on the person, without pushing help-seeking. (New)	○	○	○	○	○

* 105. The first aider should find out if there are specific reasons why the person does not want to seek professional help (e.g. concerns about finances, not having a doctor they like, or being worried they will be sent to hospital), as sometimes such reasons are based on mistaken beliefs, or can be overcome with help. (Rerate)

- Essential
- Important
- Don't know/Depends
- Unimportant
- Should not be included

* 106. Sometimes the person may need time to accept the need for treatment. The first aider should gently and respectfully persist in trying to get the person to seek help. (Rerate)

- Essential
- Important
- Don't know/Depends
- Unimportant
- Should not be included

* 107. If, in spite of the first aider's efforts, the person is still unwilling to seek or accept professional help, the first aider must respect their wishes, unless there is a risk of harm to the person or someone else. (New)

- Essential
- Important
- Don't know/Depends
- Unimportant
- Should not be included

Round 2: Updating the Mental Health First Aid Guidelines for Helping someone with Depression

What to do if there are concerns for safety

Please rate how important (from 'essential' to 'should not be included') you think it is that each statement be included in the guidelines.

Please keep our definitions in mind when responding.

* 108. If the person is at risk of harming themselves or others, the first aider should:

	Essential	Important	Depends/Don't know	Unimportant	Should not be included
a. Ask the person if it's okay to talk to someone else about their concerns for the person's safety. (Rerate)	◯	◯	◯	◯	◯
b. Tell the person that the first aider needs to get someone else involved even without their permission. (Rerate)	◯	◯	◯	◯	◯
c. Seek help on their behalf even without the person's permission. (Rerate)	◯	◯	◯	◯	◯
d. Involve the person in decisions about who else should be told about the risk of harm. (New)	◯	◯	◯	◯	◯
e. Ask the person to take steps to get help, e.g. see a GP. (New)	◯	◯	◯	◯	◯
f. Tell the person that the first aider may need to arrange professional help for them if they don't take action. (New)	◯	◯	◯	◯	◯
g. Tell the person that the first aider will check in with them at a later time. (New)	◯	◯	◯	◯	◯
h. Consider the safety of themselves and others, and take any necessary protective action, e.g. contact the police or mental health crisis team. (New)	◯	◯	◯	◯	◯

Round 2: Updating the Mental Health First Aid Guidelines for Helping someone with Depression

Thank you!

Thank you for sharing your expertise and time with us.

If anything in this survey has caused you distress and you would like to talk with someone about it you can contact the appropriate crisis help line below:

Australia: Lifeline on 13 11 14

Canada: National Suicide prevention Lifeline on 1800 273 TALK (8255)

Denmark: Suicide hotline 70 201 201

Finland: SOS Crisis Centre 010 195 202

The Netherlands: Suicide hotline 113Online

New Zealand: Lifeline Aotearoa on 0800 543 354

Republic of Ireland: Samaritans on 116 123

Sweden: Suicide hotline 020 22 00 60

UK: Samaritans on 116 123

USA: National Suicide prevention Lifeline on 1800 273 TALK (8255)

Round 3: Updating the Mental Health First Aid Guidelines for Helping someone with Depression

Information about this research

Purpose of this research

The aim of this current research project is to update the mental health first aid guidelines for how a member of the public should give assistance to a person who is experiencing depression. These guidelines are being developed for high income Western countries.

How this questionnaire was developed

The statements in this questionnaire were derived from the results of the Round 2 survey and comprise any new items that need to be rerated. An item is rerated when:

- 70%–79% of panel members from both expert groups rated it as essential or important, OR
- 70%-79% of one group rated it as essential or important and 80%-100% of the other group did.

Consent to participate

It is important for you to know that participation in this study is completely voluntary. You are not under any obligation to participate and you can withdraw at any time.

We would like to thank you for your time and effort, and encourage you to provide us with feedback on this process.

Definitions used in this survey

Mental health first aid is the help offered to a person developing a mental health problem, experiencing a worsening of an existing mental health problem, or in a mental health crisis. The first aid is given until appropriate professional help is received or until the crisis resolves.

The person refers to the person who the mental health first aider is concerned may be experiencing depression.

The first aider refers to a concerned family member, friend, work colleague or member of the community, who provides help to a person who may be experiencing depression.

Mental health professional refers to a trained health professional who is treating/working with the person. This could be a psychologist, mental health nurse or psychiatrist. It could also be another health professional who has specialist mental health training, e.g. social worker, occupational therapist or GP.

Instructions

Please complete the questionnaire by rating each statement ***according to how important you believe it is for inclusion in the guidelines*** for providing mental health first aid to a person who may be experiencing depression. Please keep in mind that the guidelines will be used by the general public. The statements need to be rated according to their importance for someone ***without a counselling or clinical background***.

This questionnaire should take approximately 10 minutes to complete. You can complete the survey in two or more sittings. **Your answers are saved when you click 'Next' at the bottom of a page.** This marks your page and you can begin again at a later date on the next page. ***Please be aware that once you have logged on and started responding you must complete the questionnaire on the same computer.***

Round 3: Updating the Mental Health First Aid Guidelines for Helping someone with Depression

Information about you

* 1. What is your name? (This allows us to determine who has completed the Round 3 survey. Your name will be deleted from your data when the project is complete).

[ ]

Overview of the study

Section 1: Learning about depression
Section 2: Approaching someone who may be experiencing depression
Section 3: Being supportive
Section 4: Communicating effectively
Section 5: Encouraging help seeking
Section 6: Encouraging self-help strategies
Section 7: What to do if they do not want help
Section 8: Concerns for safety

Round 3: Updating the Mental Health First Aid Guidelines for Helping someone with Depression

Learning about depression

Please rate how important (from 'essential' to 'should not be included') you think it is that each statement be included in the guidelines.

Please keep our definitions in mind when responding.

* 2. If assisting someone from a cultural background that is different from the first aider's, the first aider should learn about how depression symptoms may manifest in people from the person's cultural background.

- ◯ Essential
- ◯ Important
- ◯ Don't know/Depends
- ◯ Unimportant
- ◯ Should not be included

Round 3: Updating the Mental Health First Aid Guidelines for Helping someone with Depression

Approaching someone who may be experiencing depression

Please rate how important (from 'essential' to 'should not be included') you think it is that each statement be included in the guidelines.

Please keep our definitions in mind when responding.

If the first aider notices signs or symptoms of depression

* 3. If the first aider notices changes in the mood, behaviour or daily functioning of someone they know, they should approach the person about their concerns.

- Essential
- Important
- Don't know/Depends
- Unimportant
- Should not be included

Starting a conversation

* 4. The first aider should ask the person if they would like to talk to the first aider about how they are feeling.

- Essential
- Important
- Don't know/Depends
- Unimportant
- Should not be included

When talking to the person

* 5. The first aider should ask the person if anyone else knows how they are feeling.

- Essential
- Important
- Don't know/Depends
- Unimportant
- Should not be included

* 6. The first aider should not use language related to a potential diagnosis when talking with the person.

- ◯ Essential
- ◯ Important
- ◯ Don't know/Depends
- ◯ Unimportant
- ◯ Should not be included

Round 3: Updating the Mental Health First Aid Guidelines for Helping someone with Depression

Being supportive

Please rate how important (from ‘essential’ to ‘should not be included’) you think it is that each statement be included in the guidelines.

Please keep our <u>definitions</u> in mind when responding.

<u>Offering support</u>

* 7. If the first aider does not feel that they are able to help the person, they should ask someone else to take on the first aider role.

- ◯ Essential
- ◯ Important
- ◯ Don't know/Depends
- ◯ Unimportant
- ◯ Should not be included

* 8. The first aider should offer emotional support of a more positive future in whatever form the depressed person will accept.

- ◯ Essential
- ◯ Important
- ◯ Don't know/Depends
- ◯ Unimportant
- ◯ Should not be included

* 9. The first aider should let the person know that getting better takes time, but that it will happen.

- ◯ Essential
- ◯ Important
- ◯ Don't know/Depends
- ◯ Unimportant
- ◯ Should not be included

Offering practical assistance

* 10. The first aider should explore with the person how their symptoms affect their daily life.

- ◯ Essential
- ◯ Important
- ◯ Don't know/Depends
- ◯ Unimportant
- ◯ Should not be included

Round 3: Updating the Mental Health First Aid Guidelines for Helping someone with Depression

Communicating effectively

Please rate how important (from ‘essential’ to ‘should not be included’) you think it is that each statement be included in the guidelines.

Please keep our definitions in mind when responding.

Talking to the person

* 11. The first aider should use the same terminology that the person uses when discussing their experience, except if the person uses unhelpful or stigmatising language.

- ◯ Essential
- ◯ Important
- ◯ Don't know/Depends
- ◯ Unimportant
- ◯ Should not be included

* 12. Where possible, the first aider should allow the person to guide the duration of the conversation.

- Essential
- Important
- Don't know/Depends
- Unimportant
- Should not be included

Cultural considerations when communicating with the person

* 13. If cultural differences are interfering with the first aider's ability to help the person, they should talk to a mental health service that specialises in working with people from different cultural backgrounds.

- Essential
- Important
- Don't know/Depends
- Unimportant
- Should not be included

Round 3: Updating the Mental Health First Aid Guidelines for Helping someone with Depression

Help-seeking

Please rate how important (from ‘essential’ to ‘should not be included’) you think it is that each statement be included in the guidelines.

Please keep our definitions in mind when responding.

How to assist the person with help-seeking

* 14. The first aider should ask the person whether they think they would benefit from professional help.

- Essential
- Important
- Don't know/Depends
- Unimportant
- Should not be included

Preparing for the appointment with the health professional

* 15. The first aider should ask the person how much involvement they want the first aider to have with planning for and attending their appointment.

- ◯ Essential
- ◯ Important
- ◯ Don't know/Depends
- ◯ Unimportant
- ◯ Should not be included

Round 3: Updating the Mental Health First Aid Guidelines for Helping someone with Depression

Self-help strategies

Please rate how important (from ‘essential’ to ‘should not be included’) you think it is that each statement be included in the guidelines.

Please keep our definitions in mind when responding.

* 16. If the person is interested in self-help strategies, the first aider should:

	Essential	Important	Don't know/Depends	Unimportant	Should not be included
a. Discuss with them a range of self-help strategies that might be helpful	◯	◯	◯	◯	◯
b. Encourage the person to use appropriate strategies.	◯	◯	◯	◯	◯

Round 3: Updating the Mental Health First Aid Guidelines for Helping someone with Depression

What to do if there are concerns for safety

Please rate how important (from ‘essential’ to ‘should not be included’) you think it is that each statement be included in the guidelines.

Please keep our definitions in mind when responding.

* 17. If the person is at risk of harming themselves or others, the first aider should ask the person to take steps to get help, e.g. see a GP.

- ◯ Essential
- ◯ Important
- ◯ Don't know/Depends
- ◯ Unimportant
- ◯ Should not be included

Round 3: Updating the Mental Health First Aid Guidelines for Helping someone with Depression

Thank you!

Thank you for sharing your expertise and time with us.

If anything in this survey has caused you distress and you would like to talk with someone about it you can contact the appropriate crisis help line below:

Australia: Lifeline on 13 11 14

Canada: National Suicide prevention Lifeline on 1800 273 TALK (8255)

Denmark: Suicide hotline 70 201 201

Finland: SOS Crisis Centre 010 195 202

The Netherlands: Suicide hotline 113Online

New Zealand: Lifeline Aotearoa on 0800 543 354

Republic of Ireland: Samaritans on 116 123

Sweden: Suicide hotline 020 22 00 60

UK: Samaritans on 116 123

USA: National Suicide prevention Lifeline on 1800 273 TALK (8255)
